# Effects of temperature on proliferation of myoblasts from donor piglets with different thermoregulatory maturities

**DOI:** 10.1186/s12860-021-00376-4

**Published:** 2021-06-26

**Authors:** Katharina Metzger, Dirk Dannenberger, Armin Tuchscherer, Siriluck Ponsuksili, Claudia Kalbe

**Affiliations:** 1 Institute of Muscle Biology and Growth, Leibniz Institute for Farm Animal Biology (FBN), Wilhelm-Stahl-Allee 2, 18196 Dummerstorf, Germany; 2 Institute of Genome Biology, Leibniz Institute for Farm Animal Biology (FBN), Wilhelm-Stahl-Allee 2, 18196 Dummerstorf, Germany; 3 Institute of Genetics and Biometry, Leibniz Institute for Farm Animal Biology (FBN), Wilhelm-Stahl-Allee 2, 18196 Dummerstorf, Germany

**Keywords:** Satellite cells, Myoblasts, Temperature, Thermal stress, Age of donor, Pig

## Abstract

**Background:**

Climate change and the associated risk for the occurrence of extreme temperature events or permanent changes in ambient temperature are important in the husbandry of farm animals. The aim of our study was to investigate the effects of permanent cultivation temperatures below (35 °C) and above (39 °C, 41 °C) the standard cultivation temperature (37 °C) on porcine muscle development. Therefore, we used our porcine primary muscle cell culture derived from satellite cells as an in vitro model. Neonatal piglets have limited thermoregulatory stability, and several days after birth are required to maintain their body temperature. To consider this developmental step, we used myoblasts originating from thermolabile (five days of age) and thermostable piglets (twenty days of age).

**Results:**

The efficiency of myoblast proliferation using real-time monitoring via electrical impedance was comparable at all temperatures with no difference in the cell index, slope or doubling time. Both temperatures of 37 °C and 39 °C led to similar biochemical growth properties and cell viability. Only differences in the mRNA expression of myogenesis-associated genes were found at 39 °C compared to 37 °C with less MYF5, MYOD and MSTN and more MYH3 mRNA. Myoblasts grown at 35 °C are smaller, exhibit higher DNA synthesis and express higher amounts of the satellite cell marker PAX7, muscle growth inhibitor MSTN and metabolic coactivator PPARGC1A. Only permanent cultivation at 41 °C resulted in higher HSP expression at the mRNA and protein levels. Interactions between the temperature and donor age showed that MYOD, MYOG, MYH3 and SMPX mRNAs were temperature-dependently expressed in myoblasts of thermolabile but not thermostable piglets.

**Conclusions:**

We conclude that 37 °C to 39 °C is the best physiological temperature range for adequate porcine myoblast development. Corresponding to the body temperatures of piglets, it is therefore possible to culture primary muscle cells at 39 °C. Only the highest temperature of 41 °C acts as a thermal stressor for myoblasts with increased HSP expression, but it also accelerates myogenic development. Cultivation at 35 °C, however, leads to less differentiated myoblasts with distinct thermogenetic activity. The adaptive behavior of derived primary muscle cells to different cultivation temperatures seems to be determined by the thermoregulatory stability of the donor piglets.

**Supplementary Information:**

The online version contains supplementary material available at 10.1186/s12860-021-00376-4.

## Background

Climate change has caused an associated risk for the occurrence of extreme heat events in farm animals that have been subjected to heat stress [1]. In conventional pig husbandry, heat abatement plays a major role [2], whereas the challenges in free-range farming are quite different.

During birth, piglets must overcome many challenges, such as respiration, digestion, nutrition and thermoregulation, having to regulate their own body temperature to survive [3]. Adaptive thermogenesis is a specialized type of heat production and occurs in brown adipose tissue and skeletal muscle [4]. Newborn piglets do not possess brown adipose tissue [5, 6]; for this reason, they are thermolabile after birth, and an appropriate thermal environment is needed [7]. Body temperature rises to the physiological value of 39 °C within 48 h *p.p.* [8, 9], and after the first week of age, thermoregulatory functions are fully developed [10].

Satellite cells are quiescent myogenic stem cells [11]. They are involved in hypertrophic muscle growth and regeneration and maintain the muscle stem cell reservoir. The first isolation was performed by using rat muscle [12]. In this in vitro model, satellite cells develop into proliferating and differentiating progenies and are therefore a suitable model for muscle biology research. This approach provides the opportunity to directly investigate the influence of cultivation conditions such as changes in temperature. For instance, the effects of different but permanent cultivation temperatures on primary human skeletal muscle cells (range of 37 to 41 °C) and on C2C12 cells (an immortalized mouse muscle cell line, range of 35 to 41 °C) were investigated [13–15]. For avian primary muscle cell cultures, the range for permanent temperature experiments was from 33 to 43 °C (turkey [16–19], chicken [20, 21]). In pigs, the effects of heat stress during housing are already well investigated [22–24], whereas in vitro studies in muscle cell cultures are still rare. The first studies with isolated satellite cells of pigs (*M. semitendinosus* or *M. longissimus*) focused on precultivation at a control temperature and subsequent heat stress at 40.5 °C or 41 °C [25, 26].

The aim of our study was to investigate the effect of permanent cultivation temperatures below (35 °C) and above (39 °C and, 41 °C) the standard cultivation temperature (37 °C) on the proliferative growth of satellite cell progenies originating from *M. rhomboideus* of thermolabile (five days of age) and thermostable piglets (twenty days of age). We used real-time impedimetric cell growth monitoring, morphological and biochemical properties and the expression analysis of myogenesis-associated genes to characterize the temperature-dependent effects while considering the thermoregulatory maturity of the donor piglets.

## Results

### Real-time growth monitoring

**Fig. 1:**
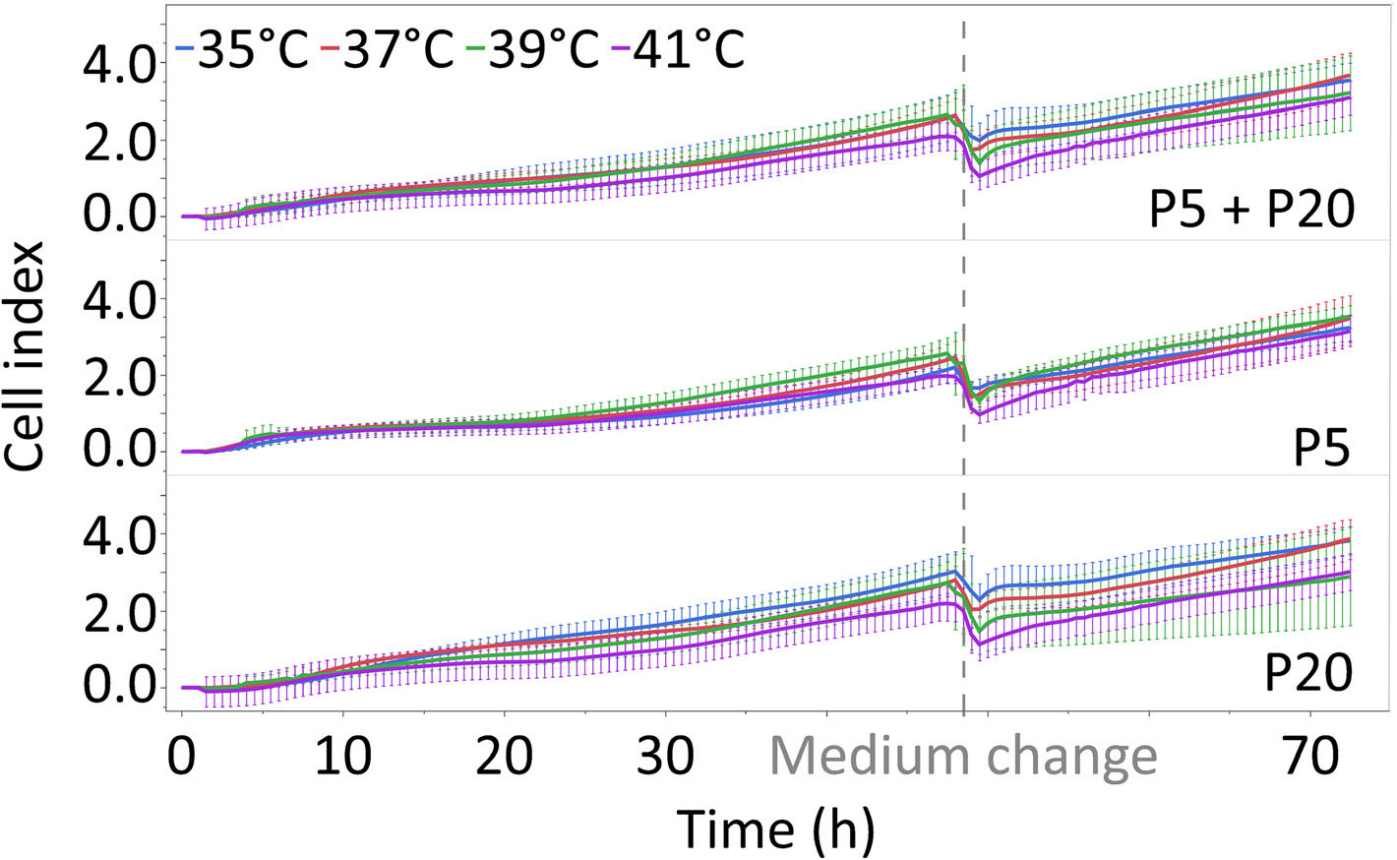
Cell indices (means ± standard deviations) measured in real time every 30 min over 72 h using the xCELLigence RTCA SP system in all myoblasts (P5 + P20), pool 5 (P5) and pool 20 (P20) that were permanently cultured at 35°, 37°, 39° or 41 °C. Values were generated from three independent experiments

Proliferative growth at 35°, 37°, 39° and 41 °C was monitored in real time with the xCELLigence RTCA SP system (Fig. 1) over a period of 72 h. The cell index (CI) was used to measure the relative change in the electrical impedance that represents the cell status. Important for the impedance are the number and the size of cells that are attached on the electrode in the bottom of the e-plate. The average CI was unaffected by the temperature (Table 1, *P* = 0.905) or pool (*P* = 0.696), with no interaction between the temperature and pool (*P* = 0.978).

**Table 1:** Average cell index parameters (least square means ± standard errors) generated from real-time monitoring of 72 h proliferative growth

Parameter	Temperature (T)				Pool (P)		T *P*	P *P*	T × P *P*
	35 °C	37 °C	39 °C	41 °C	5	20			
Cell index (arbitrary units)	1.67 ± 0.12	1.63 ± 0.12	1.58 ± 0.12	1.37 ± 0.12	1.47 ± 0.09	1.65 ± 0.09	0.905	0.696	0.978
Slope (1/h)	0.048 ± 0.004	0.045 ± 0.004	0.042 ± 0.004	0.039 ± 0.004	0.041 ± 0.002	0.045 ± 0.002	0.323	0.297	0.385
Doubling time^a^ (h)	20.4 ± 1.2	22.7 ± 1.3	20.6 ± 1.3	21.8 ± 1.3	23.3 ± 0.9	19.4 ± 0.9	0.524	0.007	0.934

^a^ The doubling time was calculated over a 67 h period (from 5 to 72 h), starting at 5 h to allow the myoblasts to attach after seeding

The doubling time (h) describes the period required to double a CI value. The doubling time was calculated over a 67 h period (from 5 to 72 h), starting at 5 h to allow the myoblasts to attach after seeding. The average doubling time was unaffected by the temperature (Table 1, *P* = 0.524). However, the average doubling time was affected by the pool, with a higher doubling time of pool 5 (*P* = 0.007), but not by the interaction of the temperature and pool (*P* = 0.934). The slope (1/h) characterizes the steepness, inclination or change of a curve. The slope was not affected by the temperature (Table 1, *P* = 0.323), pool (*P* = 0.297) or their interaction (*P* = 0.385).

### Biochemical properties of growth

In a combined assay, the DNA and protein contents (Table 2) were detected in the monolayers. The DNA content (μg/well) was equivalent to the cell number and was affected by the temperature (*P* = 0.006) but not by the pool (*P* = 0.606) or the interaction of both (*P* = 0.930). Higher contents were found at 35 °C and 39 °C than at 37 °C (*P* ≤ 0.034), with an unchained content at 41 °C (*P* ≥ 0.317). The protein content (μg/well) was unchanged between the temperatures (*P* = 0.894), pool (*P* = 0.785) or their interaction (*P* = 0.869).

**Table 2:** Biochemical properties (least square means ± standard errors) of growth after 72 h of proliferation

Parameter	Temperature (T)				Pool (P)		T *P*	P *P*	T × P *P*
	35 °C	37 °C	39 °C	41 °C	5	20			
DNA content (μg/well)	0.47 ± 0.04 ^a^	0.28 ± 0.04 ^b^	0.54 ± 0.04 ^a^	0.43 ± 0.04 ^ab^	0.44 ± 0.03	0.42 ± 0.03	0.006	0.606	0.930
Protein content (μg/well)	20.62 ± 3.34	22.57 ± 3.34	21.35 ± 3.34	24.04 ± 3.34	21.68 ± 2.36	22.61 ± 2.36	0.894	0.785	0.869
DNA synthesis (Abs 450 nm)	1.62 ± 0.07 ^a^	1.27 ± 0.07 ^b^	1.34 ± 0.07 ^ab^	1.11 ± 0.07 ^b^	1.31 ± 0.05	1.36 ± 0.05	< 0.001	0.431	0.794
LDH activity (IU/L)	41.85 ± 2.27 ^a^	24.92 ± 2.27 ^b^	22.39 ± 2.27 ^b^	26.85 ± 2.27 ^b^	26.30 ± 1.61	31.70 ± 1.61	< 0.001	0.032	0.715
TUNEL^+^ cells (%)	0.05 ± 0.01	0.02 ± 0.01	0.04 ± 0.01	0.07 ± 0.01	0.04 ± 0.01	0.04 ± 0.01	0.141	0.681	0.712

*Abs* absorbance, *LDH* lactate dehydrogenase

^a, b^ Labeled least square means within a row with different letters differ (*P* < 0.05)

The cell proliferation ELISA is based on the measurement of 5-bromo-2′-deoxyuridine (BrdU) incorporation during DNA synthesis (Table 2). DNA synthesis was affected by the temperature (*P* <  0.001), with a higher rate at 35 °C than at 37 °C and 41 °C (*P* ≤ 0.013) but not by the pool (*P* = 0.431) or the interaction of both (*P* = 0.794).

Proliferating cell nuclear antigen (PCNA, Table 3) is a proliferation marker, and mRNA expression was affected by the temperature (*P* <  0.001) but not by the pool (*P* = 0.060) or the interaction of both (*P* = 0.174). The highest mRNA expression was found at 35 °C and decreased with increasing temperature. In addition, the mRNA expression at 37 °C was increased compared to 39 and 41 °C (*P* <  0.001).

**Table 3:** Expression of genes associated with cellular development and stress (least square means ± standard errors) after 72 h of proliferation

Gene	Temperature (T)				Pool (P)		T *P*	P *P*	T × P *P*
	35 °C	37 °C	39 °C	41 °C	5	20			
PCNA mRNA	1.66 ± 0.05 ^a^	0.93 ± 0.05 ^b^	0.55 ± 0.05 ^c^	0.49 ± 0.05 ^c^	0.85 ± 0.04	0.96 ± 0.04	< 0.001	0.060	0.174
DAD1 mRNA	0.87 ± 0.05	0.78 ± 0.05	0.85 ± 0.05	0.96 ± 0.05	0.83 ± 0.04	0.89 ± 0.04	0.159	0.253	0.415
PPARGC1A mRNA	1.40 ± 0.07 ^a^	0.91 ± 0.07 ^b^	0.39 ± 0.07 ^c^	0.39 ± 0.07 ^c^	0.97 ± 0.05	0.57 ± 0.05	< 0.001	< 0.001	0.105
SORBS1 mRNA	0.66 ± 0.07 ^a^	0.91 ± 0.07 ^ab^	1.14 ± 0.07 ^b^	1.12 ± 0.07 ^b^	0.97 ± 0.05	0.94 ± 0.05	< 0.001	0.667	0.503
Heat shock proteins									
HSP25/27 mRNA	0.81 ± 0.08 ^b^	0.80 ± 0.08 ^b^	0.79 ± 0.08 ^b^	1.50 ± 0.08 ^a^	0.94 ± 0.05	1.01 ± 0.05	< 0.001	0.376	0.746
HSP70 mRNA	0.57 ± 0.08 ^b^	0.46 ± 0.08 ^b^	0.75 ± 0.08 ^b^	1.26 ± 0.08 ^a^	0.83 ± 0.06	0.68 ± 0.06	< 0.001	0.077	0.260
HSP90 mRNA	0.87 ± 0.06 ^b^	0.76 ± 0.06 ^b^	0.86 ± 0.06 ^b^	1.29 ± 0.06 ^a^	0.90 ± 0.04	0.99 ± 0.04	< 0.001	0.139	0.388
HSP70 protein	0.18 ± 0.25 ^b^	0.15 ± 0.25 ^b^	0.24 ± 0.25 ^b^	2.33 ± 0.25 ^a^	0.70 ± 0.18	0.76 ± 0.18	< 0.001	0.816	0.999
HSP90 protein	2.23 ± 0.56 ^b^	2.59 ± 0.56 ^b^	3.96 ± 0.56 ^b^	7.09 ± 0.56 ^a^	3.97 ± 0.40	3.97 ± 0.40	< 0.001	0.997	0.273
HSF1 protein	5.33 ± 1.55	4.71 ± 1.55	1.63 ± 1.55	2.17 ± 1.55	3.32 ± 1.10	3.60 ± 1.10	0.282	0.855	0.820

The mRNA expression data are expressed as arbitrary units after normalization with the endogenous reference gene RN18S

The protein expression data are expressed as arbitrary units after normalization with Coomassie staining

^a, b, c^ Labeled least square means within a row with different letters differ (*P* < 0.05)

Apoptosis was investigated using a commercial TUNEL assay (Table 2), and the mRNA expression of the gene encoding the defender against apoptotic cell death (DAD1, Table 3). There were not temperature-dependent effects on the percentage of TUNEL-positive cells and mRNA expression of DAD1 (*P* ≥ 0.141), pool-dependent effects (*P* ≥ 0.253) or the interaction between the temperature and pool on both apoptotic properties (*P* ≥ 0.415).

Lactate dehydrogenase (LDH, Table 2) is an enzyme that is ubiquitously present in all cells and will be liberated from the cell interior in the cell culture supernatant after cell damage. Significant effects of the temperature (*P* <  0.001) and pool (*P* = 0.032) were detected, but no interaction was detected between the two (*P* = 0.715). Increased LDH activity was found at 35 °C compared to all temperatures (*P* ≤ 0.002) and in pool 20 compared to pool 5 (*P* = 0.032).

### Cell viability and development

**Fig. 2:**
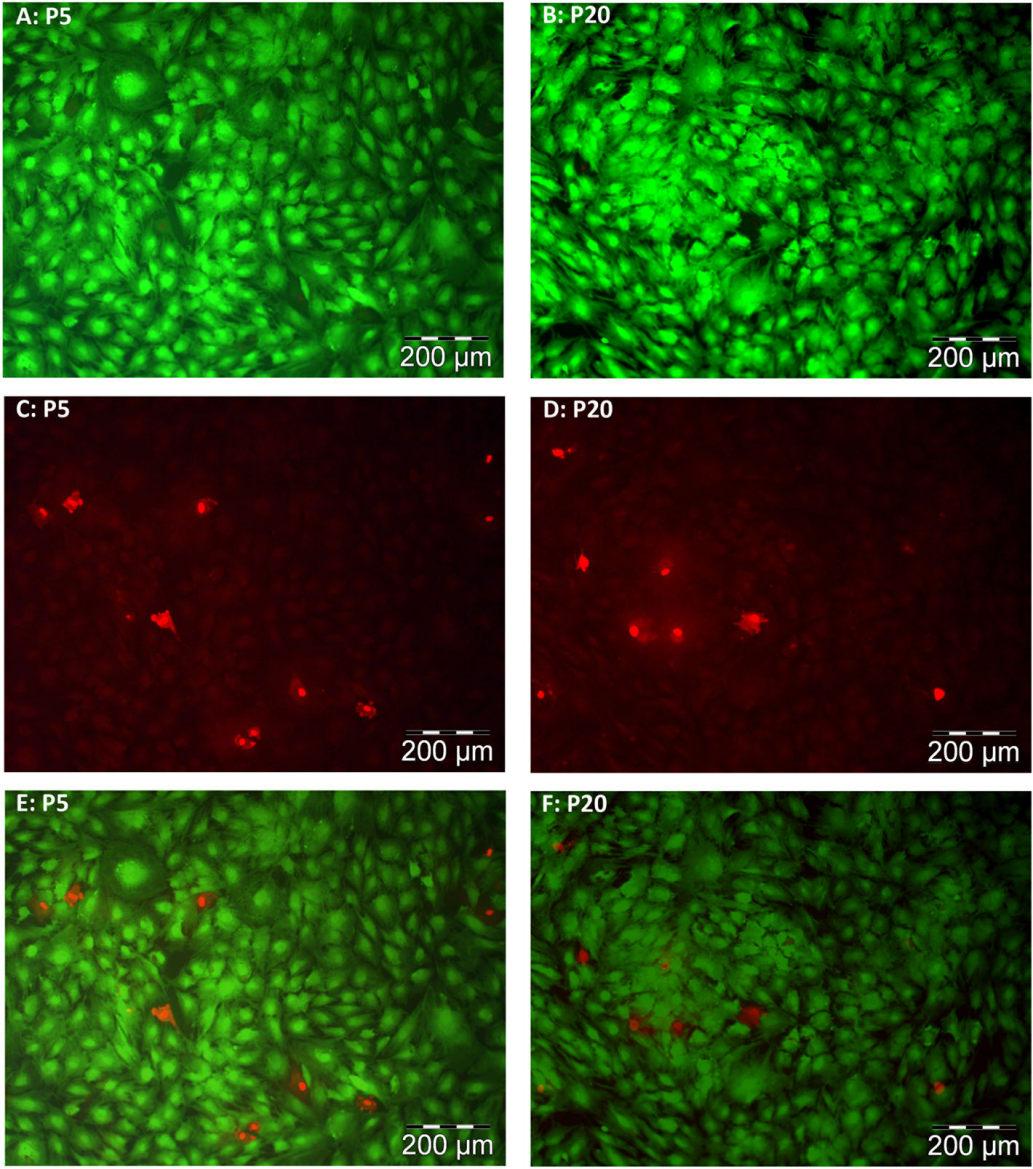
Live/dead staining was performed with fluorescein diacetate (FDA) and propidium iodide (PI) for pool 5 (P5) and pool 20 (P20). Viable cells were able to convert nonfluorescent FDA into the green fluorescent metabolite fluorescein because of esterase-dependent conversion (**A**, **B**). The nuclei staining dye PI (red) was able to pass through dead cell membranes and intercalate with the cell’s DNA double helix (**C**, **D**). An overlay of both (**E**, **F**) after 72 h of growth at 41 °C is exemplarily shown. For every pool, 30 pictures were analyzed (Nikon Microphot-SA microscope, Nikon Corporation, Tokyo, Japan; Cell^F, Olympus Corporation, Tokyo, Japan)

To evaluate the viability after 72 h of proliferative growth, combined staining was performed with fluorescein diacetate (FDI, marker for living cells) and propidium iodide (PI, marker for dead cells). The viability was affected by the temperature (*P* <  0.001) but not by the pool (*P* = 0.242) or the interaction of both (*P* = 0.551). The viability at 41 °C (92.7 ± 0.6%) was lower than that at all other temperatures (vs. 35 °C: 98.3 ± 0.6%, vs. 37 °C: 97.0 ± 0.6%, vs. 39 °C: 98.5 ± 0.6%, *P* <  0.001 each). In addition, FDI-stained myoblasts (Fig. 2) were used to determine their size. The cell size was highly affected by the temperature (*P <* 0.001) and pool (*P <* 0.001) but not by the interaction of both (*P* = 0.151). At 35 °C (1525 ± 48 μm^2^), the cell size was smaller than that at all other temperatures (vs. 37 °C: 1758 ± 48 μm^2^, vs. 39 °C: 1833 ± 48 μm^2^, vs. 41 °C: 1943 ± 48 μm^2^, *P* ≤ 0.020). The cells of pool 5 were smaller than those of pool 20 (1597 ± 34 μm^2^ vs. 1932 ± 34 μm^2^, *P* <  0.001).

**Fig. 3:**
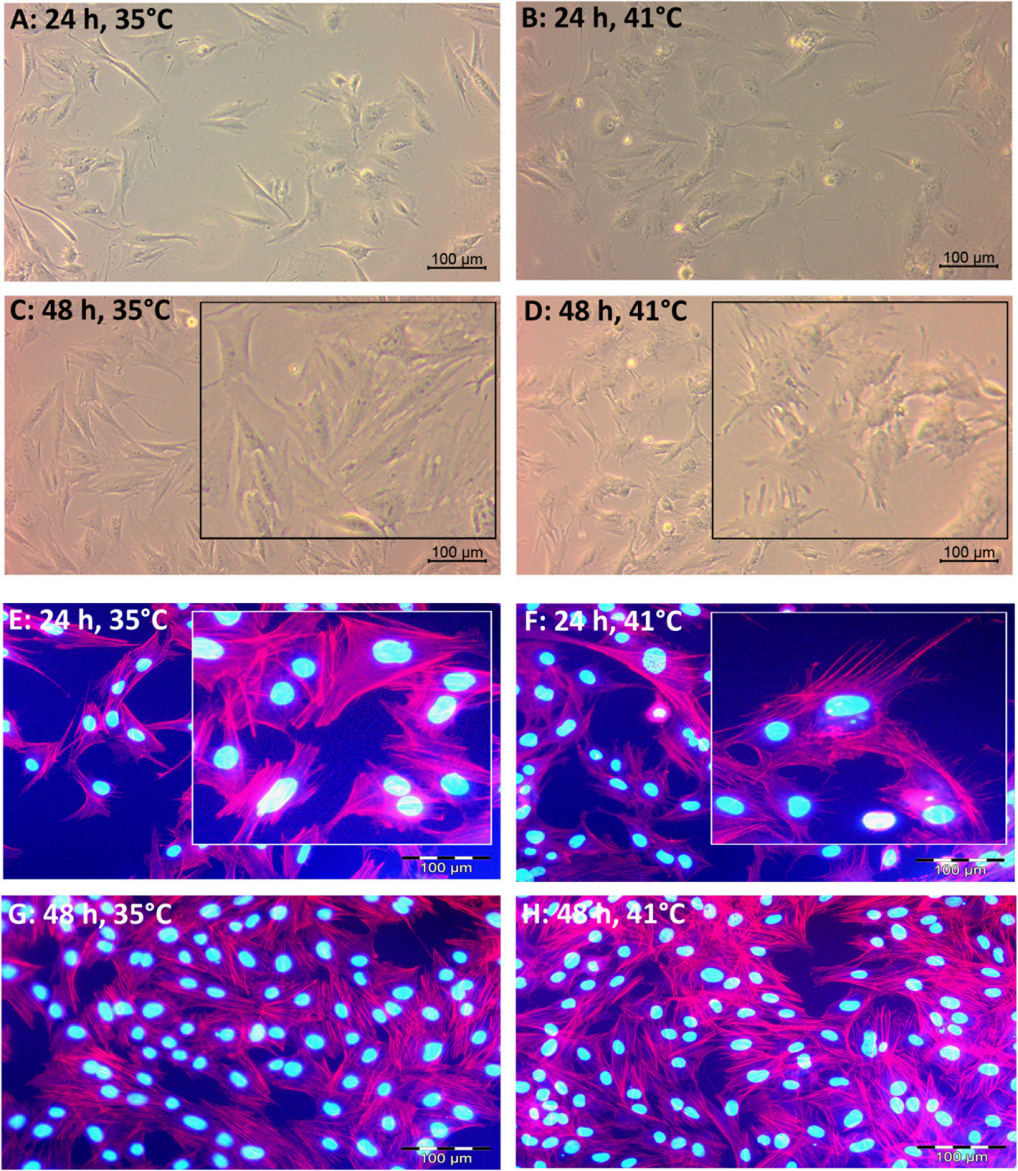
Myoblasts derived from satellite cells of *M. rhomboideus* of 5-day-old piglets were seeded on gelatin-coated dishes and permanently cultivated at 35 °C or at 41 °C for 24 h or 48 h. Images of living cells (**A**-**D**) were taken with a Primovert microscope and Axiocam ERc5s (Carl Zeiss AG, Oberkochen, Germany). A staining for actin filaments with Phalloidin CruzFluor™ 594 Conjugate (red) and 4′,6-Diamidin-2-phenylindol (DAPI) for the nuclei (blue) was performed. Images of the phalloidin and DAPI stainings were taken with Leica DM 2400 fluorescence microscope (Leica Microsystems, Wetzlar, Germany). A higher magnification was presented in the inserts

As shown in Fig. 3 (see also Additional file 1, Fig. S6), the myoblasts grown at 35 °C and 41 °C differed in their cellular shapes. Especially after 24 h and 48 h of proliferation, the myoblasts at 41 °C (Fig. 3D, F) showed finger-shaped protrusions that the cells at 35 °C (Fig. 3C, E) did not form.

**Fig. 4:**
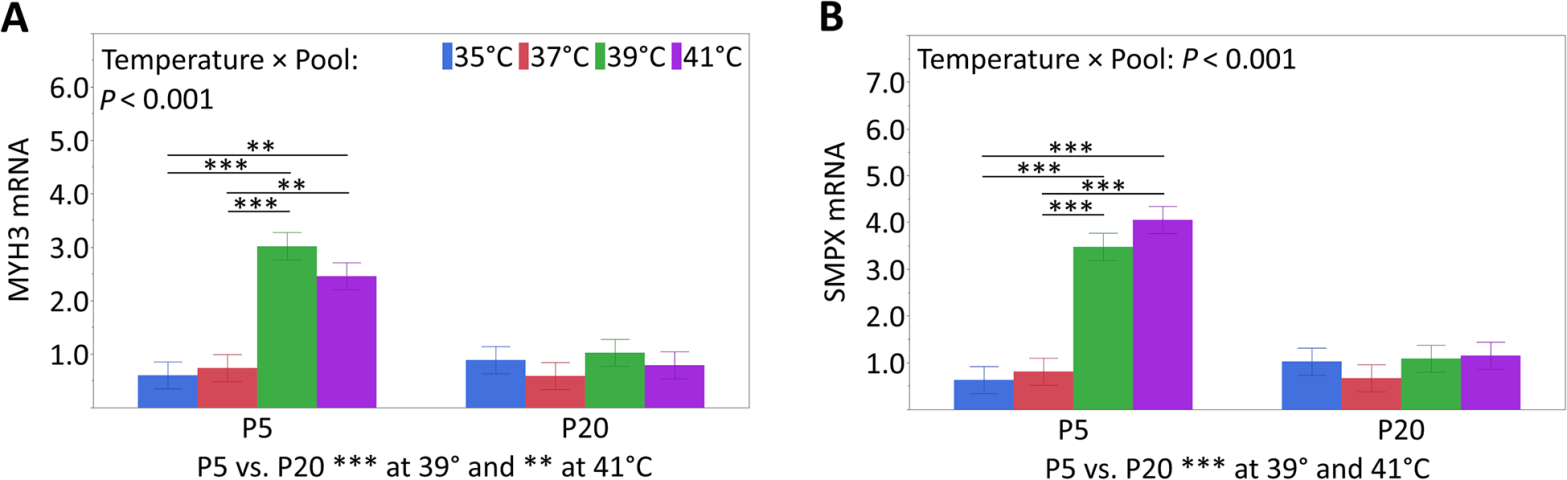
Temperature × pool interactions for MYH3 (**A**) and SMPX (**B**) mRNA expression analyzed in proliferating myoblasts of pool 5 and pool 20 after 72 h of permanent cultivation at 35°, 37°, 39° or 41 °C. Data (least square means and standard errors) are expressed as arbitrary units after normalization to RN18S expression as an endogenous reference gene. Significant differences within each pool are indicated by asterisks (***P < 0.001, ** *P* < 0.01), and significant differences between the pools are explained below

Peroxisome proliferator-activated receptor gamma coactivator 1-alpha (PPARGC1A, Table 3) is a transcriptional coactivator of energy metabolism. The expression was affected by the temperature and pool (both *P* <  0.001) but not by their interaction (*P* = 0.105). The expression decreased with increasing temperature. In addition, the expression of pool 5 was higher than that of pool 20. Sorbin and SH3 domain containing 1 (SORBS1, also known as ponsin; Table 3) is involved in growth factor-induced signal transduction, cell adhesion, and cytoskeletal organization, and its expression was affected by the temperature (*P* <  0.001) but not by the pool (*P* = 0.667) or the interaction of both (*P* = 0.503). The mRNA expression at 35 °C was lower than that at 39 °C and 41 °C (*P* <  0.001 each), whereas the expression at 35 °C and 37 °C was similar (*P* = 0.064). Myosin-3 (MYH3, also known as MyHCemb) encodes the embryonic isoform of myosin in skeletal muscle. The mRNA expression of MYH3 (Fig. 4A) was affected by the interaction between the temperature and pool (*P* <  0.001). In pool 5, the mRNA expression at 35 °C was lower than those at 39 °C and 41 °C (*P* ≤ 0.002), and the expression at 37 °C was lower than those at 39 °C and 41 °C (*P* ≤ 0.004). In pool 20, mRNA expression was not affected by the temperature (*P* ≥ 0.915). In addition, the mRNA expression at both highest temperatures was increased in pool 5 compared to pool 20 (*P* ≤ 0.005). Murine small muscle protein X-linked (SMPX, also known as CLS or Chisel) encodes a 9 kDa protein in heart and skeletal muscle cells. The mRNA expression of SMPX (Fig. 4B) displayed the same mRNA expression pattern as that described for MYH3.

### Heat shock proteins

The mRNA expression of heat shock proteins (Table 3) was affected by the temperature (*P* <  0.001) but not by the pool (*P* ≥ 0.077) or the interaction of both (*P* ≥ 0.260). Higher expression levels of HSP25/27, HSP70 and HSP90 were found at 41 °C than at 35°, 37° and 39 °C (*P* ≤ 0.002).

The protein expression (see also Additional file 1, Fig. S1-S5) of HSP70 (Table 3) and HSP90 (Table 3) was affected by the temperature (*P* <  0.001) but not by the pool (*P* ≥ 0.816) or the interaction of both (*P* ≥ 0.273). HSP70 protein expression was ten times higher at 41 °C than at all other temperatures (*P* <  0.001 each). HSP90 protein expression at 41 °C was increased in the same manner (*P* ≤ 0.002). The protein expression of heat shock factor 1 (HSF1, Table 3) was unaffected by the temperature (*P* = 0.282), pool (*P* = 0.855) or the interaction of both (*P* = 0.820).

### mRNA expression of transcription and growth factors

#### Transcription factors

**Fig. 5:**
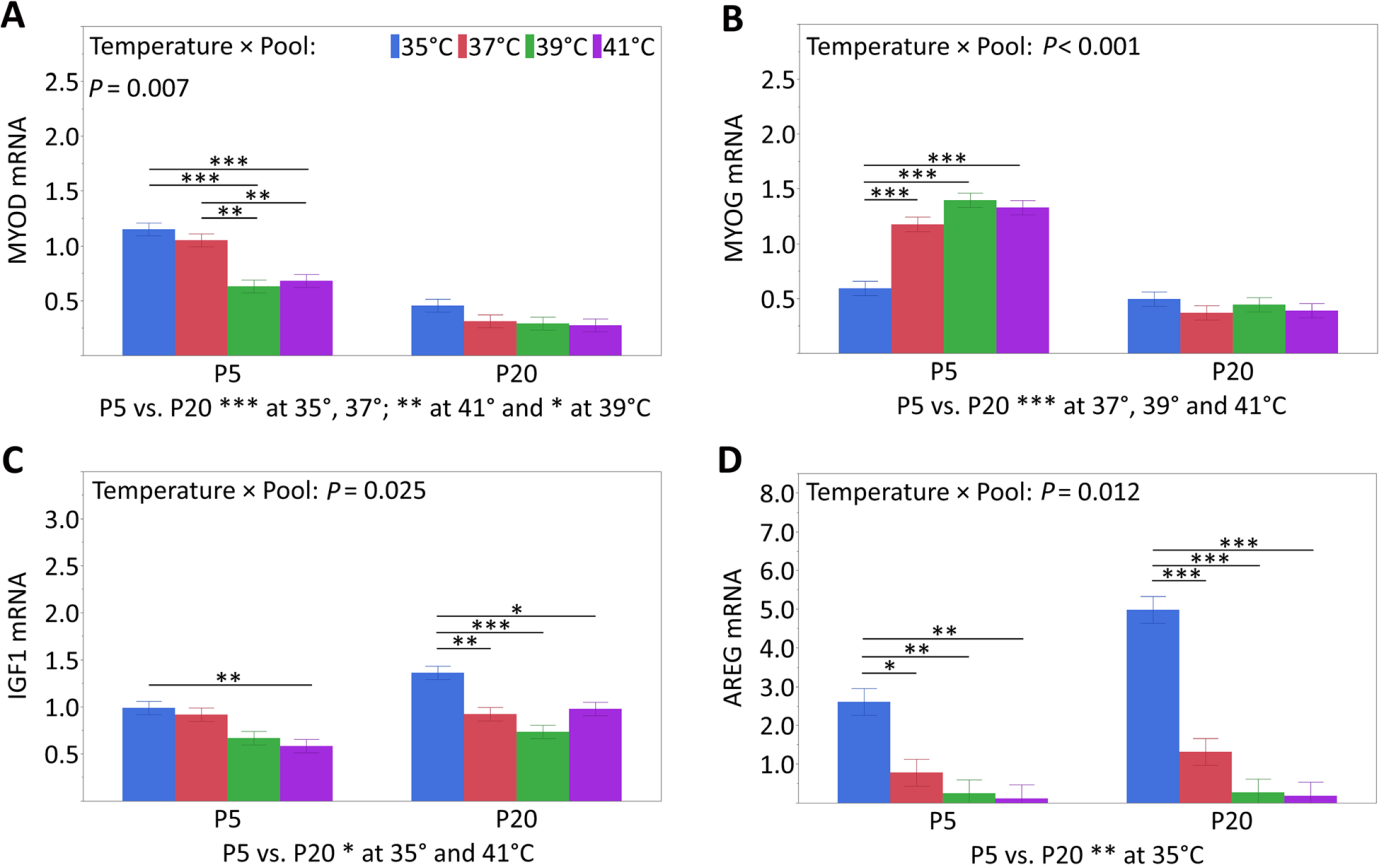
Temperature × pool interactions for MYOD (**A**), MYOG (**B**), IGF1 (**C**) and AREG (**D**) mRNA expression analyzed in proliferating myoblasts of pool 5 and pool 20 after 72 h of permanent cultivation at 35°, 37°, 39° or 41 °C. Data (least square means and standard errors) are expressed as arbitrary units after normalization to RN18S expression as an endogenous reference gene. Significant differences within each pool are indicated by asterisks (*P < 0.05, **P < 0.01 or ***P < 0.001), and significant differences between the pools at the same temperature are explained below

The mRNA expression of the satellite cell marker paired box 7 (PAX7, Table 4) and myogenic factor 5 (MYF5, Table 4) was affected by the temperature (*P* ≤ 0.022) and pool (*P* <  0.001), with no interaction between the two (*P* ≥ 0.426). PAX7 mRNA expression at 35 °C was higher than that at 41 °C (*P* = 0.024) but not at 37 °C or 39 °C (*P* ≥ 0.233). In addition, PAX7 mRNA expression was higher in pool 5 than in pool 20 (*P* <  0.001). Higher MYF5 mRNA expression was found at the lower cultivation temperatures of 35 °C and 37 °C compared to 39 °C (*P* ≤ 0.047) and 41 °C (*P* ≤ 0.009). In addition, the mRNA expression of pool 5 was higher than that of pool 20 (*P* <  0.001). The mRNA expression of myoblast determination factor (MYOD, Fig. 5A) and myogenin (MYOG, Fig. 5B) was affected by the interaction between the temperature and pool (*P* ≤ 0.007). For both genes, there were no temperature-dependent effects in pool 20. For pool 5, higher MYOD mRNA expression was found at the lower cultivation temperatures of 35 °C and 37 °C compared to 39 °C (*P* ≤ 0.002) and 41 °C (*P* ≤ 0.007). In addition, the MYOD mRNA expression of pool 5 was higher than that of pool 20 (*P* ≤ 0.015) at all temperatures. The MYOG mRNA expression of pool 5 at 35 °C was lower than that at all temperatures (*P* <  0.001), and pool 5 exhibited higher MYOG mRNA than pool 20 at 37°, 39° and 41 °C (*P* <  0.001). The mRNA expression of myogenic regulatory factor 4 (MRF4, Table 4) was unchanged by the temperature (*P* = 0.061) and the interaction of the temperature and pool (*P* = 0.291). The mRNA expression of pool 20 was higher than that of pool 5 (*P* <  0.001).

**Table 4:** mRNA expression of myogenesis-associated genes (least square means ± standard errors) after 72 h of proliferation

Gene	Temperature (T)				Pool (P)		T *P*	P *P*	T × P *P*
	35 °C	37 °C	39 °C	41 °C	5	20			
Myogenic transcription factors									
PAX7	0.92 ± 0.06 ^a^	0.76 ± 0.06 ^ab^	0.88 ± 0.06 ^ab^	0.66 ± 0.06 ^b^	0.91 ± 0.04	0.69 ± 0.04	0.022	< 0.001	0.846
MYF5	0.96 ± 0.06 ^a^	0.71 ± 0.06 ^a^	0.45 ± 0.06 ^b^	0.38 ± 0.06 ^b^	0.78 ± 0.04	0.47 ± 0.04	< 0.001	< 0.001	0.426
MRF4	1.87 ± 0.19	1.87 ± 0.19	1.67 ± 0.21	0.88 ± 0.29	1.05 ± 0.15	2.09 ± 0.17	0.061	< 0.001	0.291
Growth factors and growth factor receptors									
MSTN	0.85 ± 0.04 ^a^	0.69 ± 0.04 ^b^	0.42 ± 0.04 ^c^	0.37 ± 0.04 ^c^	0.70 ± 0.03	0.46 ± 0.03	< 0.001	< 0.001	0.233
IGF2	0.67 ± 0.07 ^b^	0.71 ± 0.07 ^b^	0.74 ± 0.07 ^ab^	1.02 ± 0.07 ^a^	1.01 ± 0.05	0.55 ± 0.05	0.015	< 0.001	0.195
EGF	0.79 ± 0.15 ^b^	0.71 ± 0.15 ^b^	1.09 ± 0.15 ^b^	2.42 ± 0.15 ^a^	1.27 ± 0.10	1.24 ± 0.10	< 0.001	0.828	0.384
IGF1R	0.89 ± 0.08 ^ab^	0.65 ± 0.08 ^b^	1.00 ± 0.08 ^a^	0.83 ± 0.08 ^ab^	0.71 ± 0.06	0.96 ± 0.06	0.043	0.006	0.082
EGFR	1.22 ± 0.10 ^ab^	0.88 ± 0.10 ^b^	1.36 ± 0.10 ^a^	1.40 ± 0.10 ^a^	1.06 ± 0.07	1.37 ± 0.07	0.009	0.008	0.165

The mRNA expression data are expressed as arbitrary units after normalization with the endogenous reference gene RN18S

^a, b, c^ Labeled least square means within a row with different letters differ (*P* < 0.05)

#### Growth factors and their receptors

The mRNA expression of myostatin (MSTN, Table 4), a negative regulator of muscle growth, was affected by the temperature (*P* <  0.001) and pool (*P* <  0.001), with no interaction between the two (*P* = 0.233). With increasing temperature, mRNA expression decreased (*P* ≤ 0.035), but there were no differences between the highest temperatures (39 °C vs. 41 °C, *P* = 0.810). In addition, the mRNA expression of pool 5 was higher than that of pool 20 (*P* <  0.001).

The mRNA expression of insulin-like growth factor 1 (IGF1, Fig. 5C) was affected by the interaction of the temperature and pool (*P* = 0.025). In pool 5, the mRNA expression at 35 °C was higher than that at 41 °C (*P* = 0.017), whereas in pool 20, the mRNA expression was higher at 35 °C than at all other temperatures (*P* ≤ 0.026). Moreover, the mRNA expression of pool 5 was lower than that of pool 20 but only at the most extreme temperatures (35 °C and 41 °C, *P* ≤ 0.032). The mRNA expression of insulin-like growth factor 2 (IGF2, Table 4) was affected by the temperature (*P* = 0.015) and pool (*P* <  0.001) with no effect on the interaction of both (*P* = 0.195). The mRNA expression levels were higher at 41 °C than at 35 °C and 37 °C (*P* ≤ 0.037) as well as in pool 5 compared to pool 20 (*P* < 0.001). The temperature-dependent mRNA expression of epidermal growth factor (EGF, Table 4, *P* < 0.001) was found with a two-fold increase at 41 °C compared to 35°, 37° and 39 °C (*P* < 0.001). EGF-specific mRNA remained unchanged by the pool (*P* = 0.828) and the interaction of the temperature and pool (*P* = 0.384). The mRNA expression of amphiregulin (AREG, Fig. 5D), another ligand of EGF receptor (EGFR), was affected by the interaction of the temperature and pool (*P* = 0.012). For both pools, the mRNA expression at 35 °C was higher than that at all other temperatures (*P* < 0.030). In addition, the mRNA expression at 35 °C for pool 5 was lower than that for pool 20 (*P* = 0.003). The mRNA expression levels of insulin-like growth factor 1 receptor (IGF1R, Table 4) and EGFR (Table 4) were affected by the temperature (*P* ≤ 0.043) and pool (*P* ≤ 0.008) but not by the interaction between the two (*P* ≥ 0.082). The lowest IGF1R mRNA expression was found at 37 °C, which was different from 39 °C (*P* = 0.030), whereas EGFR mRNA was lower at 37 °C than that at 39 °C and 41 °C (*P* ≤ 0.020). In addition, less IGF1R and EGFR mRNA was expressed in pool 5 than in pool 20 (*P* ≤ 0.008).

## Discussion

The perinatal period is characterized by drastic impacts of the climatic and nutritional environments in piglets [27]. In addition, newborn piglets are naturally exposed to cold but do not possess brown adipose tissue [5, 6] and are unable to maintain their body temperature in the first week of life [10].

Heat stress has a negative impact on livestock production and may influence the growth, animal health and welfare [2, 28, 29]. Known results of environmental hyperthermia in pigs are decreased feed intake, increased heat load, oxidative stress and endotoxemia, among others [24, 30, 31].

Previous studies with porcine satellite cells investigated heat shock conditions after precultivation at 37 °C followed by a single high temperature stimulus over a fixed period [25, 26]. For avian satellite cell cultures, a temperature range was considered that included the body temperature and heat stress-inducing temperatures [16–21]. Based on this, our porcine primary muscle cells were permanently cultured between 35 °C and 41 °C with 2 °C incremental temperatures. The standard cultivation temperature for porcine muscle cells is 37 °C, and the temperature challenge with 2 °C below and 2 °C and 4 °C above the standard cultivation temperature is moderate but continually present. To the best of our knowledge, this is the first study of a permanent temperature regime used for porcine satellite cell-derived primary muscle cultures.

### Effects under permanent cultivation at physiological temperatures

Regarding the standard cultivation temperature (37 °C) of porcine primary muscle cell cultures and the body temperature of piglets (39 °C), we could not detect any differences using real-time monitoring of the proliferative growth over 72 h. Moreover, the expression of HSPs, as markers for cellular stress [32], remained unchanged. Therefore, both temperatures seem to be physiological temperatures for porcine primary cells [33], and some studies preferentially cultivated porcine primary cells at a euthermic temperature of 39 °C [34, 35]. The missing effects on the majority of biochemical properties, such as DNA synthesis or LDH and apoptotic properties, also confirmed similar proliferative growth. However, we found effects on the mRNA expression of myogenesis-associated genes between 37 °C and 39 °C that argue for differences in the proliferative growth and differentiation potential at the molecular level. Obviously, the differences were insufficient to induce adaptive changes in the proliferative growth and cellular phenotype. Especially in the case of the early muscle regulatory factors (MRF) MYF5 and MYOD [36], there seems to be a temperature threshold between 37 °C and 39 °C for the reduction of the amount of mRNA as a sign for terminating of proliferation. This fits the higher DNA content and lower PCNA mRNA of muscle cells at 39 °C compared to those at 37 °C. PCNA is a cofactor of DNA polymerase δ whose levels correlate with DNA synthesis, reaching a maximum during the S-phase [37, 38]. In agreement, culturing primary pig cells at 39 °C enhances cellular processes such as hyperplasia or earlier entry in the differentiation of stromal-vascular cells [39]. The increased mRNA expression of MYH3 and SMPX also indicates differential processes before myotube formation. The latter is known to promote cell signaling-dependent myocyte fusion and cytoskeletal dynamics [40]. Moreover, in mice, it was shown that the SMPX gene responded to biomechanical stress [41].

### Effects under permanent cultivation at cold temperature

To the best of our knowledge, this is the first attempt to study the effects of permanent cultivation at temperatures below the physiological range on primary myoblasts. Only studies with turkey or chicken muscle cells used lower temperatures down to 33 °C compared to the control of 38 °C during proliferation but after precultivation at control temperatures [16–19, 21]. We found no studies using mammalian primary muscle cells and temperatures below organismic body temperature or usually used cultivation temperatures. Cultivation below the physiological temperature was possible without consequences for the proliferative growth behavior monitored in real time in our study. In addition, due to HSP expression, we conclude that 35 °C did not act as a stressor for myoblasts.

However, we found a reduced size of our myoblasts at 35 °C compared to the other (higher) temperatures. Together with the increased DNA synthesis, DNA content and higher amount of PCNA mRNA, this argues for increased proliferative activity, which should normally be detectable by the impedance-based real-time monitoring of the cells. However, we believe that the increased LDH activity as an indication of more damaged and lysed myoblasts could be the reason for the compensation of the effect on CI and the CI-derived parameter slope and doubling time. Due to the comparable myoblast viability at 35 °C and at physiological temperatures of approximately 98%, the increased LDH activity seems to be more a sign of higher proliferative activity than of cell damage at low temperatures. Moreover, we found that the temperature did not affect the apoptotic capability of myoblasts, as formerly shown for chicken myoblasts [20]. With respect to the mRNA expression of myogenesis-associated genes, it seems that myoblasts cultivated at 35 °C are closer to activated satellite cells and less differentiated (in terms of premyoblasts) than comparable cells cultured at higher temperatures. Higher mRNA expression levels of the satellite cell marker PAX7 and MYF5 and MYOD also suggest a greater proximity to ancestor satellite cells than to mature myoblasts described after heat stress cultivation (see below). The highest mRNA expression of the PPARGC1A gene at 35 °C argues for the potential thermogenetic activity of muscle cells due to the colder cultivation temperatures. PPARGC1A is known as a transcriptional coactivator involved in mitochondriogenesis and mitochondrial energy metabolism [42], and the role of muscle tissue as a location for thermogenesis was studied in animals without brown adipose tissue, such as pigs (reviewed in [4]). For instance, the skeletal muscle of 5-day-old piglets contributes 97% of cold-induced whole-body heat production [43]. The reduced mRNA expression of genes involved in differentiation, such as SMPX and MYOG, and in muscle cell structures, such as MYH3, fits with the less differentiated state of myoblasts. The mRNA expression of the EGFR ligand AREG was higher at 35 °C than at the other temperatures. Increased AREG expression was also detected after hypothermia in healthy rat prostate tissue [44]. Although the role of specific EGFR ligands in skeletal muscle growth is not clear, EGF could stimulate skeletal muscle growth and differentiation in vitro [45, 46].

### Effects under permanent heat stress cultivation

Our HSP expression results at the mRNA and protein levels clearly showed that 41 °C was sufficient to induce a heat shock response. This is consistent with the results for cultivation at 40.5 °C [25] and 41 °C [26] in porcine muscle cells after precultivation at 37 °C. The unaffected HSF1 expression highlights a two-component feedback loop in which HSF1 positively regulates HSP70, whereas HSP70 negatively regulates HSF1 [47].

In our study, the proliferative ability of satellite cells seemed to end after 72 h at 41 °C. This result is in agreement with other studies using muscle cells derived from Langtang swine [26] or derived from chicks that were reared at 5 °C higher than standard conditions [48]. A rather opposite phenotype of the myoblasts at 41 °C was observed compared to that at 35 °C. Cultivation at 41 °C led to less viability, although the viability after 72 h of cultivation was still approximately 93%. The involvement of apoptosis in heat stress responses of myoblasts is unclear; both our studies and poultry studies [20, 48] did not find effects on the apoptotic capability, whereas Gao et al. [26] found increased apoptosis. The cell size at 41 °C was increased, which was also seen by Gao et al. [26]. Larger cells argue for a higher degree of myoblast differentiation, as described for myocytes [49]. This finding fits with lower MYF5 and MYOD mRNA levels and higher MYH3 mRNA expression at higher temperatures (39 °C and 41 °C). As discussed in the section about physiological temperatures, there seems to be a threshold between 37 °C and 39 °C leading to changes in the mRNA expression. This result is in agreement with lower MSTN mRNA expression at higher temperatures in our study because MSTN (or growth and differentiation factor 8, GDF8) is a negative stimulator of skeletal muscle growth [50]. Higher expression at 41 °C was also found for the growth factors IGF2 and EGF. Generally, IGF2 and EGF stimulate both the proliferation and differentiation of muscle cells [45, 46, 51]; reviewed in [52, 53]. We formerly showed that in our porcine muscle cells, IGF2 and EGF mRNA expression increased from proliferating myoblasts to differentiating myotubes [54]. Even the higher SORBS1 expression at 41 °C supported a higher degree of differentiation of myoblasts because SORBS1 is known to be expressed shortly after the onset of myogenic differentiation [55, 56] and marks the establishment of costameres, the cell–matrix contacts. Another protein that is associated with the costameric cytoskeleton, SMPX, was increased during proliferation at higher temperatures (39 °C and 41 °C). This result is in agreement with the hypomethylation of SMPX, indicating upregulated gene expression in pig skeletal muscle after constant heat stress at a 30 °C vs. 22 °C housing temperature [57]. Moreover, in C2C12 cells, it was shown that SMPX expression is related to the formation of pseudopodia [41]. Pseudopodia are arm-like protrusions filled with the cytoplasm of eukaryotic cells and are used for locomotion. Known types of pseudopodia in muscle cells are lamellipodia and filopodia. Myoblast elongation during differentiation is accompanied by the dynamic extension of filopodia composed of actin filaments to contact neighboring muscle cells before fusion (reviewed in [58]). The increased cell size together with the finger-like protrusions (Fig. 3) at 41 °C argue for a higher degree of myoblast differentiation and a prestage of myoblast fusion.

### Effect of the age of the donor piglets

The pools differed in the piglet age at satellite cell isolation by only 15 days of age. However, the peri/neonatal period of piglets is very important for their development. At the age of five days, piglets are thermolabile, whereas those at twenty days of age maintain their body temperature independently [3]. In addition, the muscular alteration of piglets during birth and the first three weeks of age is significant. We formerly showed that the total fiber number in *M. semitendinosus* was not fixed at birth. The postnatal increase in the myofiber number may be related to both the elongation of existing muscle fibers (hypertrophy) and genesis of tertiary myofibers (hyperplasia, [59]). To our knowledge, this is the first study to consider piglets of different ages in satellite cell isolation, thus accounting for the thermoregulation capacity of donor animals. The effects of temperature were formerly investigated in muscle cell cultures derived from satellite cells of different muscle types in chickens [21] or from turkey muscle with different growth rates [16].

In the current study, we found that both pools differed with regard to most properties, such as the doubling time, cell size and mRNA expression of the majority of myogenesis-associated genes. The MRFs (MYOD, MYF5 and MYOG), which are responsible for myogenic determination or fusion initiation, were expressed at higher mRNA levels in pool 5 than in pool 20, whereas the negative regulator of myogenesis MSTN was expressed in the opposite manner. This speaks for a closer proximity of pool 5 cells to the original satellite cells and because PAX7 was expressed in higher amounts in pool 5. This is in line with other studies on porcine satellite cells that have indicated higher PAX7 mRNA expression after birth and a decrease in the following days [60, 61]. We have also postulated the closer proximity to satellite cells for myoblasts cultured at 35 °C. Moreover, the smaller size and the higher mRNA expression of PPARGC1A in pool 5 myoblasts are in agreement with the myoblasts at 35 °C and argue for the less differentiated cells with distinct thermogenetic activity.

Significant interactions between temperature and pool were present for six of the investigated genes. For the growth factors, IGF1 (Fig. 5C) and AREG (Fig. 5D) temperature-dependent mRNA expression effects were found in both pools 5 and 20. In contrast, the four myogenesis-associated genes MYOD (Fig. 5A), MYOG (Fig. 5B), MYH3 (Fig. 4A) and SMPX (Fig. 4B) were temperature-dependently expressed only in myoblasts from pool 5 but not from pool 20. Apparently, the primary muscle cell cultures isolated from the satellite cells of the thermostable donors seem to be less responsive to the temperature changes or even thermal stress for porcine myoblasts, as shown in our study. On the other hand, primary muscle cell cultures isolated from piglets that were labile with respect to their body temperature showed a greater susceptibility to exceeding and falling below a physiological cultivation temperature.

## Conclusion

In our study, we used permanent cultivation temperatures above (39 °C and 41 °C) and below (35 °C) the standard cultivation temperature (37 °C) for primary muscle cells of pigs. The porcine muscle cells were able to comparably proliferate at all temperatures regarding their real-time monitored growth behavior. Only the highest cultivation temperature of 41 °C acted as an environmental stressor for the myoblasts, whereas temperatures 2 degrees below and above the standard temperature of 37 °C were not able to induce the expression of HSPs. From our study, we conclude that we consider both temperatures, 37 °C and 39 °C, as physiological temperatures for porcine myoblast growth and differentiation. This result is made plausible by the pigs’ body temperature of 39 °C. Cultivation below the standard temperature leads to myoblasts, which are closer to activated satellite cells and less differentiated myoblasts with thermogenetic activity in addition to myogenic determination. Cultivation above physiological temperatures leads to thermal stress in porcine myoblasts and to an acceleration of myogenic development. Myoblasts at 41 °C are larger and have a higher degree of differentiation and finger-like protrusions as a prestage of myoblast fusion. Looking at the age of the cell donor piglets, the adaptive behavior of our primary muscle cells to temperature seems to be determined. Myoblasts derived from satellite cells from thermostable donors at 20 days of age seem to be less responsive to temperature changes or thermal stress than corresponding cells derived from 5-day-old piglets, which are known as thermolabile.

## Methods

### Cell culture

The isolation of the satellite cells, the establishment of the two cell pools and their validation were carried out as described in detail by Metzger et al. [62].

In brief, two pools were established from *M. rhomboideus* of 10 female German Landrace piglets at days 5 or 20 of age (pool 5 or pool 20), aliquoted (2 × 10^6^ cells per vial) and stored in liquid nitrogen. *M. rhomboideus* is a mixed muscle with a very high proportion (approximately 75%) of oxidative fibers [63]. The muscle is located in the area of the neck and the shoulder blades and is involved in posture by supporting the head. The percentage of myogenic cells was determined by immunostaining for desmin (D1033, Sigma-Aldrich, Taufkirchen, Germany) with 98 ± 1% desmin-positive cells for pool 5 and 95 ± 2% for pool 20.

Proliferative growth was studied over 72 h, including a medium change after 48 h at 35°, 37°, 39° or 41 °C with 37 °C as the standard cultivation temperature. For each temperature, three independent experiments were performed using growth medium (DMEM (Biochrom, Berlin, Germany) supplemented with 0.2 M L-glutamine (Carl Roth, Karlsruhe, Germany), 100 IU/mL penicillin (Biochrom), 100 μg/mL streptomycin (Biochrom), 2.5 μg/mL amphotericin (Sigma-Aldrich), 10% FBS (Sigma-Aldrich), and 10% donor horse serum (HS; Sigma-Aldrich)). For real-time monitoring (e-plate 96, ACEA Biosciences Inc., San Diego, USA), the determination of DNA synthesis (96-well MP, Corning, Wiesbaden, Germany), DNA and protein contents (96-well microplate, Sarstedt, Nümbrecht, Germany), and LDH activity (96-well microplate, Sarstedt), 4000 cells per well from each pool were seeded in 10 gelatin-coated wells. For RNA and protein isolation and live/dead staining and phalloidin staining, 1 × 10^6^ cells were seeded on a gelatin-coated 100-mm cell culture dish (Sarstedt).

### Real-time monitoring of proliferation

The real-time monitoring of proliferation was carried out with the xCELLigence RTCA SP system (ACEA Biosciences Inc.). Proliferative growth was monitored over 72 h by recording the impedance every 30 min. The data are presented as the CI (arbitrary units), which corresponds to changes in impedance. For the CI, slope and doubling time data were generated with RTCA Software 1.2.1 (ACEA Biosciences Inc.) as described by [62].

### RNA isolation, reverse transcription and real-time PCR

RNA isolation, reserve transcription and real-time PCR procedures were previously described [54, 64] and were performed after 72 h of proliferative growth. Primer information is listed in Table S1 (see Additional file 1, [65–71]). Data are expressed as arbitrary units after normalization with the endogenous reference gene 18S ribosomal RNA (RN18S). RN18S expression was unaffected by the temperature (*P* = 0.121), pool (*P* = 0.281) or their interaction (*P* = 0.656).

### Protein isolation and western blot

After 72 h of proliferative growth, the plates were washed with phosphate-buffered saline (PBS, Biochrom). Then, 400 μL of homogenization buffer at a of pH 7.0 (100 mM 2-[4-(2-hydroxyethyl)-1-piperazinyl] ethanesulfonic acid (Carl Roth), 250 mM sucrose (Carl Roth), 4 mM disodium ethylenediaminetetraacetate (Carl Roth), 1 mM dithiothreitol (Carl Roth), and protease inhibitor stock solution according to the manufacturer’s instructions (complete Mini, Roche, Mannheim, Germany)) was added and incubated on ice for 5 min. Next, the cells were scraped, collected in a preparation tube (Sarstedt), placed in an ultrasonic bath (Omnilab, Bremen, Germany) of cold water for 2 min, and subsequently centrifuged at 14,000 g and 4 °C for 10 min. The protein content was determined at 280 nm by using the microplate reader Synergy™ MX (BioTek, Bad Friedrichshall, Germany) and the microvolume plate Take 3 (BioTek). The supernatants were aliquoted and stored at − 80 °C.

Aliquots of 100 μL of proliferated cells were defrosted. Afterwards, a denaturing protein sample pretreatment was performed by adding blue loading buffer (Cell Signaling Technology, Boston, USA), at 94 °C 4 min. The proteins were separated in a Maxi Buffer tank with gel running buffer (0.12 mol 2-amino-2-hydroxymethyl-propane-1,3-diol (Tris), 0.97 mol glycine and 0.02 mol SDS) with an electrophoresis constant power supply (Consort, Turnhout, Belgium). Subsequently, proteins were transferred (60 min, 1.0 mA/cm^2^) to a PVDF membrane (Carl Roth) with a semidry blotting unit (Peqlab/VWR, Darmstadt, Germany). The SDS-PAGE run time was 1.5 h at 125 mA. Thereafter, the blot was dried at room temperature. Nonspecific binding sites were blocked with skim milk powder in TBST (w/v = 5%) for 1 h at room temperature. The PVDF membrane was incubated with specific primary antibodies against heat shock factor 1 (HSF1, 80 kDa, 12,972, Cell Signaling Technology, Denver, USA), heat shock protein 90 (HSP90, 90 kDa, 60,318–1, Proteintech®, St. Leon-Rot, Germany) and heat shock protein 70 (HSP70, 70 kDa, sc-66,048, Santa Cruz Biotechnology, Dallas, USA) in skim milk powder in TBST (w/v = 5%). The incubation with antibodies was performed with a 1:600 dilution and completed at 6 °C overnight. After washing, the PVDF membrane was treated with the secondary antibody rabbit TrueBlot® anti-rabbit IgG HRP (18–8816, Rockland Immunochemicals, Limerick, USA) in case of HSF1 and mouse TrueBlot® Ultra anti-mouse Ig HRP (18–8817-30, Rockland Immunochemicals) for HSP90 and HSP70 at dilutions of 1:50000, respectively, for 90 min of incubation at room temperature, followed by washing three times with TBST and water. For visualization, membranes were developed with SuperSignal® West FEMTO chemiluminescent agent (Thermo Scientific, Schwerte, Germany). Membranes were scanned with a chemiluminescence imager (Intas Science Imaging Instruments, Goettingen, Germany), and band intensities were densitometrically evaluated using LabImage 1D L340 Electrophoresis Software (Kapelan Bio-Imaging, Leipzig, Germany). Western blot analyses were performed once per antibody, and samples of triplicate samples from every temperature of both pools were added per gel (see Additional file 1, Fig. S1). Equal loading of the gels and proper transfer of the proteins to the membranes were verified by Coomassie staining. For staining, the membrane was incubated for 15 min with a solution of methanol (50%, Carl Roth), acetic acid (7%, Carl Roth) and Coomassie Brilliant Blue R (0.1%, Sigma-Aldrich), and a scan followed. The membrane was then removed twice for 5 min (destain solution 1: 50% methanol, 7% acetic acid; destain solution 2: 90% methanol, 10% acetic acid). Data are expressed as the normalized protein abundance after normalization with one band after Coomassie staining (see Additional file 1, Fig. S2). The protein expression of this band was unaffected by the temperature (*P* = 0.952), pool (*P* = 0.423) or their interaction (*P* = 0.817).

### DNA synthesis

DNA synthesis was determined after 72 h of proliferative growth by using a commercial colorimetric assay Cell Proliferation ELISA, BrdU (Roche, Mannheim, Germany). The DNA synthesis assay was performed according to Palin et al. [72], measured by using Synergy™ MX (BioTek), and data are given as the absorbance at 450 nm.

### DNA- and protein contents

A combined assay of the DNA and protein contents was established by [73] and adapted for piglet myoblasts by [46]. DNA and protein contents were measured after 72 h of proliferative growth and were given as μg/well in the monolayers.

### Lactate dehydrogenase (LDH) activity

The lactate dehydrogenase (LDH) activity was measured after 72 h of proliferative growth in cell culture supernatants according to the method of Legarnd et al. [74] as modified by Mau et al. [75]. After 72 h, supernatants were collected in a preparation tube (Sarstedt) and stored at −80 °C until determination. The LDH activity was expressed as IU/mL of supernatant, the enzyme activity, which converts 1 μM NADH/min/L to NAD at 25 °C.

### Apoptosis

The percentage of TUNEL positive cells was detected after 72 h of proliferative growth using the commercial In Situ Cell Death Detection Kit, Fluorescin (Roche). The kit was used according to the manufacturer’s instructions, and the TUNEL positive cells were detected with a Leica DM 2400 fluorescence microscope (Leica Microsystems, Wetzlar, Germany) using green and blue fluorescence filters.

### Live/dead staining and cell size

After 72 h of proliferative growth, live/dead staining was performed. For this purpose, stock solutions of FDA (5 mg of FAD (Sigma-Aldrich) in 1 mL of acetone (≥ 99.9%, Carl Roth)) and PI (2 mg of PI (Carl Roth) in 1 mL of PBS (Biochrom)) were needed. The freshly prepared staining solution containing 10 mL of PBS (Biochrom), 16 μL of FDA (5 mg/mL) and 100 μL of PI (2 mg/mL) was added to cells washed two times. Then, the cells were incubated in the dark at room temperature for 5 min, washed again and analyzed with a Nikon Microphot-SA microscope (Nikon Corporation, Tokyo, Japan) using green and blue fluorescence filters. From every 100-mm cell culture dish, 30 images were taken so that in total 1080 pictures were analyzed. To analyze the cell size, images of green FDA-stained myoblasts were analyzed. From every repetition of each pool, 600 myoblasts were analysed; in total, 14,040 myoblasts were mapped with Cell^F (Olympus Corporation, Tokyo, Japan).

### Phalloidin staining

After 24 h, 48 h and 72 h of proliferative growth, cells were fixed with a solution of 4% paraformaldehyde (Carl Roth) in PBS (Biochrom) for 60 min and stored at −80 °C until the staining was performed. For the staining the cultured dishes were defrosted, washed twice with PBS (Biochrom), incubated in the dark at room temperature for 60 min with Phalloidin CruzFluor™ 594 Conjugate (actin filaments, Santa Cruz, Heidelberg, Germany) 1:1000 in PBS (Biochrom) containing 1% bovine serum albumin (Sigma-Aldrich), washed again and for counterstaining ROTI®Mount FluorCare DAPI (nuclei, Carl Roth) was added. Images of the phalloidin (red) and DAPI (blue) stainings were taken with Leica DM 2400 fluorescence microscope (Leica Microsystems, Wetzlar, Germany).

### Statistical analyses

For statistical analysis, data were subjected to analysis of variance using the MIXED procedure in SAS (Version 9.4, SAS Inst Inc., Cary, USA). The donor piglet age (pool 5 or pool 20), temperature (35°, 37°, 39° or 41 °C), the replication of the experiment (1, 2 or 3) and the interaction of temperature and pool were used as fixed factors. The experiment revealed no significance for all parameters. Differences between the least square means were analyzed with Tukey-Kramer tests. The statistical significance was defined for *P* < 0.05.

To ensure a consistent presentation of the results, we have presented the six parameters of the study that showed significant interactions between the fixed factors temperature and pool in figures. For all other parameters, the results are given in tables.

## Supplementary Information

**Additional file 1: Table S1:** Primers used for qPCR. **Fig. S1.** Overview of Western blot analysis of HSP70, HSP90 and HSF1 of myoblasts from pool 5 (5) or pool 20 (20) permanently cultured at 35°, 37°, 39° and 41 °C. Experiment was performed three times (Experiment 1, 2, 3). For quality assurance the original blots are shown individually in Fig. S3 for HSP70, in Fig. S4 for HSP90 and in Fig. S5 for HSF1. **Fig. S2:** Coomassie blue loading control staining used for normalization of Western blot analysis (Fig. S1). Myoblasts from pool 5 (5) or pool 20 (20) were permanently cultured at 35°, 37°, 39° and 41 °C. Experiment was performed three times (Experiment 1, 2, 3). **Fig. S3** Original Western blot of HSP70 of myoblasts from pool 5 (5) or pool 20 (20) permanently cultured at 35°, 37°, 39° and 41 °C. Experiment was performed three times (Experiment 1, 2, 3). **Fig. S4** Original Western blot of HSP90 of myoblasts from pool 5 (5) or pool 20 (20) permanently cultured at 35°, 37°, 39° and 41 °C. Experiment was performed three times (Experiment 1, 2, 3). **Fig. S5** Original Western blot of HSF1 of myoblasts from pool 5 (5) or pool 20 (20) permanently cultured at 35°, 37°, 39° and 41 °C. Experiment was performed three times (Experiment 1, 2, 3). **Fig. S6** Myoblasts derived from satellite cells of *M. rhomboideus* of 5-day-old piglets were seeded on gelatin-coated dishes and cultivated at 35 °C (A-F) or 41 °C (G-L) for 24 h, 48 h and 72 h. A staining for actin filaments with Phalloidin CruzFluor™ 594 Conjugate (red: A-C, G-I) alone and an overlay with 4′,6-Diamidin-2-phenylindol (DAPI) for the nuclei (blue) were shown (D-F, J-L). Images were taken with Leica DM 2400 fluorescence microscope (Leica Microsystems, Wetzlar, Germany).

## Data Availability

The datasets analysed during the current study are available from the corresponding author on reasonable request.

## References

[1] Horton RM, Mankin JS, Lesk C, Coffel E, Raymond C (2016). A review of recent advances in research on extreme heat events. Curr Clim Change Rep.

[2] St-Pierre NR, Cobanov B, Schnitkey G (2003). Economic losses from heat stress by US livestock industries. J Dairy Sci.

[3] Herpin P, Damon M, Le Dividich J (2002). Development of thermoregulation and neonatal survival in pigs. Anim Prod Sci.

[4] Fuller-Jackson JP, Henry BA (2018). Adipose and skeletal muscle thermogenesis: studies from large animals. J Endocrinol.

[5] Trayhurn P, Temple NJ, Van Aerde J (1989). Evidence from immunoblotting studies on uncoupling protein that brown adipose tissue is not present in domestic pig. Can J Physiol Pharmacol.

[6] Herpin P, Lossec G, Schmidt I, Cohen-Adad F, Duchamp C, Lefaucheur L, Goglia F, Lanni A (2002). Effect of age and cold exposure on morphofunctional characteristics of skeletal muscle in neonatal pigs. Pflüger's Arch Eur J Physiol.

[7] Tuchscherer M, Puppe B, Tuchscherer A, Tiemann U (2000). Early identification of neonates at risk: traits of newborn piglets with respect to survival. Theriogenology..

[8] Mount L (1968). The climatic physiology in the pig.

[9] Berthon D, Herpin P, Duchamp C, Dauncey MJ, Le Dividich J (1993). Modification of thermogenic capacity in neonatal pig by changes in thyroid status during late gestation. J Dev Physiol.

[10] Curtis SE, Rogler JC (1970). Thermoregulatory sympathetic ontogeny and adipokinetic in piglets: responses to cold. Am J Phys.

[11] Mauro A (1961). Satellite cell of skeletal muscle fibers. J Biophys Biochem Cytol.

[12] Bischoff R (1974). Enzymatic liberation of myogenic cells from adult rat muscle. Anat Rec.

[13] Yamaguchi T, Suzuki T, Arai H, Tanabe S, Atomi Y (2010). Continuous mild heat stress induces different mammalian myoblast, shifting fiber type from fast to slow. Am J Physiol Cell Physiol.

[14] Guo Q, Miller D, An H, Wang H, Lopez J, Lough D, He L, Kumar A (2016). Controlled heat stress promotes myofibrillogenesis during myogenesis. PLoS One.

[15] Sajjanar B, Siengdee P, Trakooljul N, Liu X, Kalbe K, Wimmers K, Ponsuksili S (2019). Cross-talk between energy metabolism and epigenetics during temperature stress response in C2C12 myoblasts. Int J Hyperth.

[16] Clark DL, Coy CS, Strasburg GM, Reed KM, Velleman SG (2016). Temperature effect on proliferation and differentiation of satellite cells from turkeys with different growth rates. Poult Sci.

[17] Clark DL, Strasburg GM, Reed KM, Velleman SG (2017). Influence of temperature and growth selection on Turkey pectoralis major muscle satellite cells adipogenic gene expression and lipid accumulation. Poult Sci.

[18] Reed KM, Mendoza KM, Abrahante JE, Barnes NE, Velleman SG, Strasburg GM (2017). Response of Turkey muscle satellite cells to thermal challenge. I Transcriptome effects in proliferating cells. BMC Genomics.

[19] Reed KM, Mendoza KM, Strasburg GM, Velleman SG (2017). Response of Turkey muscle satellite cells to thermal challenge. II Transcriptome effects in differentiating cells. Front Physiol.

[20] Harding LH, Clark DL, Halvey O, Coy CS, Yahav S, Velleman SG (2015). The effect of temperature on apoptosis and adipogenesis on skeletal muscles satellite cells derived from different muscle types. Physiol Rep.

[21] Harding LH, Halevy O, Yahav S, Velleman SG (2016). The effect of temperature on proliferation and differentiation of chicken skeletal muscle satellite cells isolated from different muscle types. Physiol Rep..

[22] Le Bellego L, van Milgen J, Noblet J (2002). Effect of high temperature and low-protein on the performance of growing-finishing pigs. J Anim Sci.

[23] Patience JF, Umboh JF, Chaplin RK, Nyachoti CM (2005). Nutritional and physiological responses of growing pigs exposed to a diurnal pattern of heat stress. Livest Prod Sci.

[24] Hao Y, Feng Y, Yang P, Feng J, Lin H, Gu X (2014). Nutritional and physiological responses of finishing pigs exposed to a permanent heat exposure during three weeks. Arch Anim Nutr.

[25] Kamanga-Sollo E, Pampusch MS, White ME, Hathaway MR, Dayton WR (2011). Effects of heat stress on proliferation, protein turnover, and abundance of heat shock protein messenger ribonucleic acid in cultured porcine muscle satellite cells. J Anim Sci.

[26] Gao C, Zhao Y, Li H, Sui W, Yan H, Wang X (2015). Heat stress inhibits proliferation, promotes growth, and induces apotosis in cultured Langtang swine skeletal muscle satellite cells. J Zhejiang Univ Sci B.

[27] Schmidt I, Herpin P (1998). Carnitine palmitoyltransferase I (CPT I) activity and its regulation by Malonyl-CoA are modulated by age and cold exposure in skeletal muscle mitochondria from newborn pigs. J Nutr.

[28] Baumgard LH, Rhoads RP (2013). Effects of heat stress on postabsorptive metabolism and energetics. Annu Rev Anim Biosci.

[29] Koch F, Thom U, Albrecht E, Weikard R, Nolte W, Kuhla B, Kuehn C (2019). Heat stress directly impairs gut integrity and recruits distinct immune cell populations into the bovine intestine. Proc Natl Acad Sci U S A.

[30] Pearce SC, Lonergan SM, Huff-Lonergan E, Baumgard LH, Gabler NK (2015). Acute heat stress and reduced nutrient intake alter intestinal proteomic profile and gene expression in pigs. PLoS One.

[31] Ganesan S, Summers CM, Pearce SC, Gabler NK, Valentine RJ, Baumgard LH, Rhoads RP, Selsby JT (2018). Short-term heat stress altered metabolism and insulin signaling in skeletal muscle. J Anim Sci.

[32] Lewis S, Handy RD, Cordi B, Billinghurst Z, Depledge MH (1999). Stress proteins (HSP’s): methods of detection and their use as an environmental biomarker. Ecotoxicology..

[33] Williams KJ, Picou AA, Kish SL, Giraldo AM, Godke RA, Bondioli KR (2008). Isolation and characterization of porcine adipose tissue-derived adult stem cells. Cells Tissues Organs.

[34] Shim H, Gutiérrez-Adán A, Chen LR, BonDurant RH, Behboodi E, Anderson GB (1997). Isolation of pluripotent stem cells from cultured porcine primordial germ cells. Biol Reprod.

[35] Xue B, Li Y, He Y, Wei R, Sun R, Yin Z, Bou G, Liu Z (2016). Porcine pluripotent stem cells derived from IVF embryos contribute to chimeric development *in vitro*. PLoS One.

[36] Rudnicki MA, Jaenisch R (1995). The MyoD family of transcription factors and skeletal myogenesis. Bioessays..

[37] Bravo R, Grank R, Blundell PA, Macdonald-Bravo H (1987). Cyclin/PCNA is the auxiliary protein of DNA polymerase-d. Nature..

[38] Baserga R (1991). Growth regulation of the PCNA gene. J Cell Sci.

[39] Bohan AE, Purvis KN, Bartosh JL, Brandebourg TD (2014). The proliferation and differentiation of primary pig preadipocytes is suppressed when cultures are incubated at 37 °Celsius compared to euthermic conditions in pigs. Adipocyte..

[40] Palmer S, Groves N, Schindeler A, Yeoh T, Biben C, Wang CC, Sparrow DB, Barnett L, Jenkins NA, Copeland NG, Koentgen F, Mohun T, Harvey RP (2001). The small muscle-specific protein Csl modifies cell shape and promotes myocyte fusion in an insulin-like growth factor 1–dependent manner. J Cell Biol.

[41] Schindeler A, Lavulo L, Harvey R (2005). Muscle costameric protein, chisel/Smpx, associates with focal adhesion complexes and modulates cell spreading in vitro via a Rac1/p38 pathway. Exp Cell Res.

[42] Quesnel H, Pére MC, Louveau I, Lefaucheur L, Perruchot MH, Prunier A, Pastorelli H, Meunier-Salaün MC, Gardan-Salmon D, Merlot E, Gondret F (2019). Sow environment during gestation: part II. Influence on piglet physiology and tissue maturity at birth. Animal..

[43] Lossec G, Lebreton Y, Hulin JC, Fillaut M, Herpin P (1998). Age-related changes in oxygen and nutrient uptake by hindquarters in newborn pigs during cold-induced shivering. Exp Physiol.

[44] Kaija H, Pakanen L, Kortelainen ML, Porvari K (2015). Hypothermia and rewarming induce gene expression and multiplication of cells in healthy rat prostate tissue. PLoS One.

[45] Roe JA, Baba AS, Harper JM, Buttery PJ (1995). Effects of growth factors and gut regulatory peptides on nutrient uptake in ovine muscle cell cultures. Comp Biochem Physiol A Physiol.

[46] Mau M, Kalbe C, Wollenhaupt K, Nürnberg G, Rehfeldt C (2008). IGF-I- and EGF-dependent DNA synthesis of porcine myoblasts is influenced by the dietary isoflavones genistein and daidzein. Domest Anim Endocrinol.

[47] Krakowiak J, Zheng X, Patel N, Feder ZA, Anandhakumar J, Valerius K, Gross DS, Khalil AS, Pincus D (2018). Hsf1 and Hsp70 constitute a two-component feedback loop that regulates the yeast heat shock response. eLife.

[48] Piestun Y, Patael T, Yahav S, Velleman SG, Halevy O (2017). Early posthatch thermal stress affects breast muscle development and satellite cell growth and characteristics in broilers. Poult Sci.

[49] Ganassi M, Badodi S, Quiroga HPO, Zammit PS, Hinits Y, Hughes SM (2018). Myogenin promotes myocyte fusion to balance fibre number and size. Nat Commun.

[50] McPherron AC, Lawler AM, Lee SJ (1997). Regulation of skeletal muscle mass in mice by a new TGF-beta superfamily member. Nature..

[51] Harper JMM, Buttery PJ (1995). Muscle cell growth. Meat Focus Int.

[52] Florini JR, Ewton DZ, Coolican SA (1996). Growth hormone and the insulin-like growth factor system in myogenesis. Endocr Rev.

[53] Oksbjerg N, Gondret F, Vestergaard M (2004). Basic principles of muscle development and growth in meat-producing mammals as affected by the insulin-like growth factor (IGF) system. Domest Anim Endocrinol.

[54] Kalbe C, Mau M, Rehfeldt C (2008). Developmental changes and the impact of isoflavones on mRNA expression of IGF-I receptor, EGF receptor and related growth factors in porcine skeletal muscle cell cultures. Growth Hormon IGF Res.

[55] Gehmlich K, Pinotsis N, Hayess K, van der Ven PF, Milting H, El Banayosy A, Korfer R, Wilmanns M, Ehler E, Fürst DO (2007). Paxillin and ponsin interact in nascent costameres of muscle cells. J Mol Biol.

[56] Gehmlich K, Hayess K, Legler C, Haebel S, Van der Ven PFM, Ehler E, Fürst DO (2010). Ponsin interacts with Nck adapter proteins: implications for a role in cytoskeletal remodelling during differentiation of skeletal muscle cells. Eur J Cell Biol.

[57] Hao Y, Cui Y, Gu X (2016). Genome-wide DNA methylation profiles changes associated with constant heat stress in pigs as measured by bisulfite sequencing. Sci Rep.

[58] Pavlath GK (2010). Spatial and functional restriction of regulatory molecules during mammalian myoblast fusion. Exp Cell Res.

[59] Bérard J, Kalbe C, Lösel D, Tuchscherer A, Rehfeldt C (2011). Potential sources of early-postnatal increase in myofibre number in pig skeletal muscle. Histochem Cell Biol.

[60] Caliaro F, Maccatrozzo L, Toniolo L, Reggiani C, Mascarello F, Patruno M (2005). Myogenic regulatory factors expressed during postnatal hyperplastic growth in porcine muscles. Basic Appl Myol.

[61] Lösel D, Tuchscherer A, Kalbe C (2013). Age-related changes in the expression of myogenesis-associated genes in the pig muscle. J Anim Sci.

[62] Metzger K, Tuchscherer A, Palin MF, Ponsuksili S, Kalbe C (2020). Establishment and validation of cell pools using primary muscle cells derived from satellite cells of piglet skeletal muscle. In Vitro Cell Dev Biol –Anim.

[63] Lösel D, Franke A, Kalbe C (2013). Comparison of different skeletal muscles from growing domestic pigs and wild boars. Arch Anim Breed.

[64] Kalbe C, Zebunke M, Lösel D, Brendle J, Hoy S, Puppe B (2018). Voluntary locomotor activity promotes myogenic growth potential in domestic pigs. Sci Rep.

[65] Lin J, Barb CR, Kraeling RR, Rampacek GB (2001). Developmental changes in the long form leptin receptor and related neuropeptide gene expression in the pig brain. Biol Reprod.

[66] Kennedy TG, Brown KD, Vaughan TJ (1994). Expression of genes for the epidermal growth factor receptor and its ligands in porcine oviduct and endometrium. Biol Reprod.

[67] Maak S, Wicke M, Swalwe HH (2005). Analysis of gene expression in specific muscles of swine and Turkey. Arch Ani Breed.

[68] Rehfeldt C, Lefaucher L, Block J, Stabenow B, Pfuhl R, Otten W, Metges CC, Kalbe C (2012). Limited and excess protein intake of pregnant gilts differently affects body composition and cellularity of skeletal muscle and subcutaneous adipose tissue of newborn and weanling piglets. Eur J Nutr.

[69] Da Costa N, Blackley R, Alzuherri H, Chang KC (2002). Quantifying the temporospatial expression of postnatal porcine skeletal myosin heavy chain genes. J Histochem Cytochem.

[70] Patruno M, Caliaro F, Maccatrozzo L, Sacchetto R, Martinello T, Toniolo L, Reggiani C, Mascarello F (2008). Myostatin shows a specific expression pattern in pig skeletal and extraocular muscles during pre- and post-natal growth. Differentiation..

[71] Jacobs K, Rohrer G, Van Poucke M, Piumi F, Yerle M, Bartenschlager H, Mattheeuws M, Van Zeveren A, Peelman LJ (2006). Porcine PPARGC1A (peroxisome proliferative activated receptor gamma coactivator 1A): coding sequence, genomic organization, polymorphisms and mapping. Cytogenet Genome Res.

[72] Palin MF, Lapointe J, Gariépy C, Beaudry D, Kalbe C (2020). Characterisation of intracellular molecular mechanisms modulated by carnosine in porcine myoblasts under basal and oxidative stress conditions. PLoS One.

[73] Rehfeldt C, Walther K (1997). A combined assay for DNA, protein and incorporated [3H] label in cultured muscle cells. Anal Biochem.

[74] Legrand C, Bour JM, Jacob C, Capiaumont J, Martial A, Marc A, Wudtke M, Kretzmer G, Demangel C (1992). Lactate dehydrogenase (LDH) activity of the cultured eukaryotic cells as marker of the number of dead cells in the medium. J Biotechnol.

[75] Mau M, Kalbe C, Viergutz T, Nürnberg G, Rehfeldt C (2008). Effects of dietary isoflavones on proliferation and DNA integrity of myoblasts derived from newborn piglets. Pediatr Res.

