# Supplementary material for: Effects of temperature on proliferation of myoblasts from donor piglets with different thermoregulatory maturities
Source: BMC Mol Cell Biol. 2021 Jun 26;22:36. doi: 10.1186/s12860-021-00376-4 (PMC8236195; doi:10.1186/s12860-021-00376-4)
Supplement: Supplementary file 1 — Additional file 1: Table S1: Primers used for qPCR. Fig. S1. Overview of Western blot analysis of HSP70, HSP90 and HSF1 of myoblasts from pool 5 (5) or pool 20 (20) permanently cultured at 35°, 37°, 39° and 41 °C. Experiment was performed three times (Experiment 1, 2, 3). For quality assurance the original blots are shown individually in Fig. S3 for HSP70, in Fig. S4 for HSP90 and in Fig. S5 for HSF1. Fig. S2: Coomassie blue loading control staining used for normalization of Western blot analysis (Fig. S1). Myoblasts from pool 5 (5) or pool 20 (20) were permanently cultured at 35°, 37°, 39° and 41 °C. Experiment was performed three times (Experiment 1, 2, 3). Fig. S3 Original Western blot of HSP70 of myoblasts from pool 5 (5) or pool 20 (20) permanently cultured at 35°, 37°, 39° and 41 °C. Experiment was performed three times (Experiment 1, 2, 3). Fig. S4 Original Western blot of HSP90 of myoblasts from pool 5 (5) or pool 20 (20) permanently cultured at 35°, 37°, 39° and 41 °C. Experiment was performed three times (Experiment 1, 2, 3). Fig. S5 Original Western blot of HSF1 of myoblasts from pool 5 (5) or pool 20 (20) permanently cultured at 35°, 37°, 39° and 41 °C. Experiment was performed three times (Experiment 1, 2, 3). Fig. S6 Myoblasts derived from satellite cells of M. rhomboideus of 5-day-old piglets were seeded on gelatin-coated dishes and cultivated at 35 °C (A-F) or 41 °C (G-L) for 24 h, 48 h and 72 h. A staining for actin filaments with Phalloidin CruzFluor™ 594 Conjugate (red: A-C, G-I) alone and an overlay with 4′,6-Diamidin-2-phenylindol (DAPI) for the nuclei (blue) were shown (D-F, J-L). Images were taken with Leica DM 2400 fluorescence microscope (Leica Microsystems, Wetzlar, Germany). [file 12860_2021_376_MOESM1_ESM.pdf]

# Effects of temperature on proliferation of myoblasts from donor piglets with different thermoregulatory maturities

Katharina Metzger[1,2], Dirk Dannenberger[1], Armin Tuchscherer[3], Siriluck Ponsuksili[2], Claudia Kalbe[1*]

[1]Institute of Muscle Biology and Growth, [2]Institute of Genome Biology, [3]Institute of Genetics and Biometry, Leibniz Institute for Farm Animal Biology (FBN), 18196 Dummerstorf, Germany

* Corresponding author: kalbe@fbn-dummerstorf.de (C. Kalbe)

**Table S1**

Primers used for qPCR.

| Gene | Accession no. or reference | Forward primer | Reverse primer | Size (bp) | $T_A$ (°C) |
|---|---|---|---|---|---|
| RN18S | [69] | ACTGAGGCCATGATTAAG | GCTATCAATCTGTCAATCC | 400 | 60 |
| AREG | NM_214376 | GCCATTGCTGCTTTTGTCTCTGCC | TGGCAGTGACCCCGATCTGCT | 198 | 60 |
| DAD1 | NM_213944 | CATCCTGCACCTCGTTGTA | CCATGTGTCCAGCAAACTGA | 140 | 60 |
| EGF | [70] | TCTGAACCCGGACGGATTTG | GACATCGCTCGCGAACGTAG | 202 | 60 |
| EGFR | [54] | TGGAGAAGCTCCCAACCA | CTCTTAATTCCTTGATAGCCACAG | 161 | 60 |
| HSP90 | [25] | CGCTGAGAAAGTGACCGTTATC | ACCTTTGTTCCACGACCCATAG | 126 | 60 |
| HSP70 | [25] | GTGGCTCTACCCGCATCCC | GCACAGCAGCACCATAGGC | 114 | 60 |
| HSP25/27 | [25] | AGGAGCGGCAGGATGAG | GGACAGGGAGGAGGAGA | 101 | 60 |
| IGF1 | [54] | CTCTTCGCATCTCTTCTACTTGGC | CCTGTGGGCTTGTTGAAATAAAA | 150 | 60 |
| IGF2 | [54] | TGGCATCGTGGAAGAGTG | AGGTGTCATAGCGGAAGAAC | 164 | 57 |
| IGF1R | [54]. | GATTCAGGCCACCTCTCTCTCC | CCCTCCTACTATCAACAGAACGGC | 139 | 60 |
| MRF4 | [71] | CGCCATCAACTACATCGAGAGGT | ATCACGAGCCCCCTGGAAT | 189 | 60 |
| MSTN | [71] | CCCGTCAAGACTCCTACAACA | CACATCAATGCTCTGCCAA | 141 | 62 |
| MYF5 | [72] | CCTGAATGCAACAGCCCT | CGGAGTTGCTGATCCGAT | 152 | 60 |
| MYH3 | [73] | CCCGGCTTTGGTCTGATTT | GGTGTCGGCTGAGAGTCA | 74 | 58 |
| MYOD | [72] | GGTGACTCAGACGCATCCA | ATAGGTGCCGTCGTAGCAGT | 108 | 60 |
| MYOG | [72] | CAACCAGGAGGAGCGAGAC | AGGGTCAGCTGTGAGCAGAT | 161 | 64 |
| PAX7 | [74] | CAACCACATCCGCCACAA | TCTTGGAGACACAGCCATGG | 101 | 58 |
| PCNA | DQ473295 | ACGCTAAGGGCAGAAGATAATGCAG | CGTGCAAATTCACCAGAAGGCATC | 173 | 60 |
| PPARGC1A | [75] | CCTGCATGAGTGTGTGCTCT | CTCAGAGTCCTGGTTGCACA | 107 | 59 |
| SMPX | NM_001078687 | CGATGGGAGCCTTTCGGCCA | GACCGCAGGTCCTGGGAGTTT | 143 | 62 |
| SORBS1 | XM_001924661 | CCACTGCAAGCCCTCAGCCTT | GTGACTCTTCGCTGCTGGGCT | 106 | 65 |

$T_A$, annealing temperature; RN18S - 18S ribosomal RNA; AREG - amphiregulin; DAD1 - dolichyl-diphosphooligosaccharide-protein glycosyltransferase subunit; EGF – epidermal growth factor; EGFR - epidermal growth factor receptor; HSP90, 70, 25/27 – heat shock protein 90, 70, 25/27; IGF1, 2 – insulin-like growth factor 1, 2; IGF1R - insulin-like growth factor 1 receptor; MRF4 - muscle-specific regulatory factor 4; MSTN – myostatin; MYF5 – myogenic factor 5; MYH3 – Myosin heavy chain-3; MYOD - myogenic determination factor; MYOG – myogenin; PAX7 – paired box 7 transcription factor; PCNA - proliferating cell nuclear antigen; PPARGC1A - peroxisome proliferator-activated receptor gamma coactivator 1-alpha, SMPX - small muscle protein x-linked; SORBS1 – sorbin and SH3 domain containing

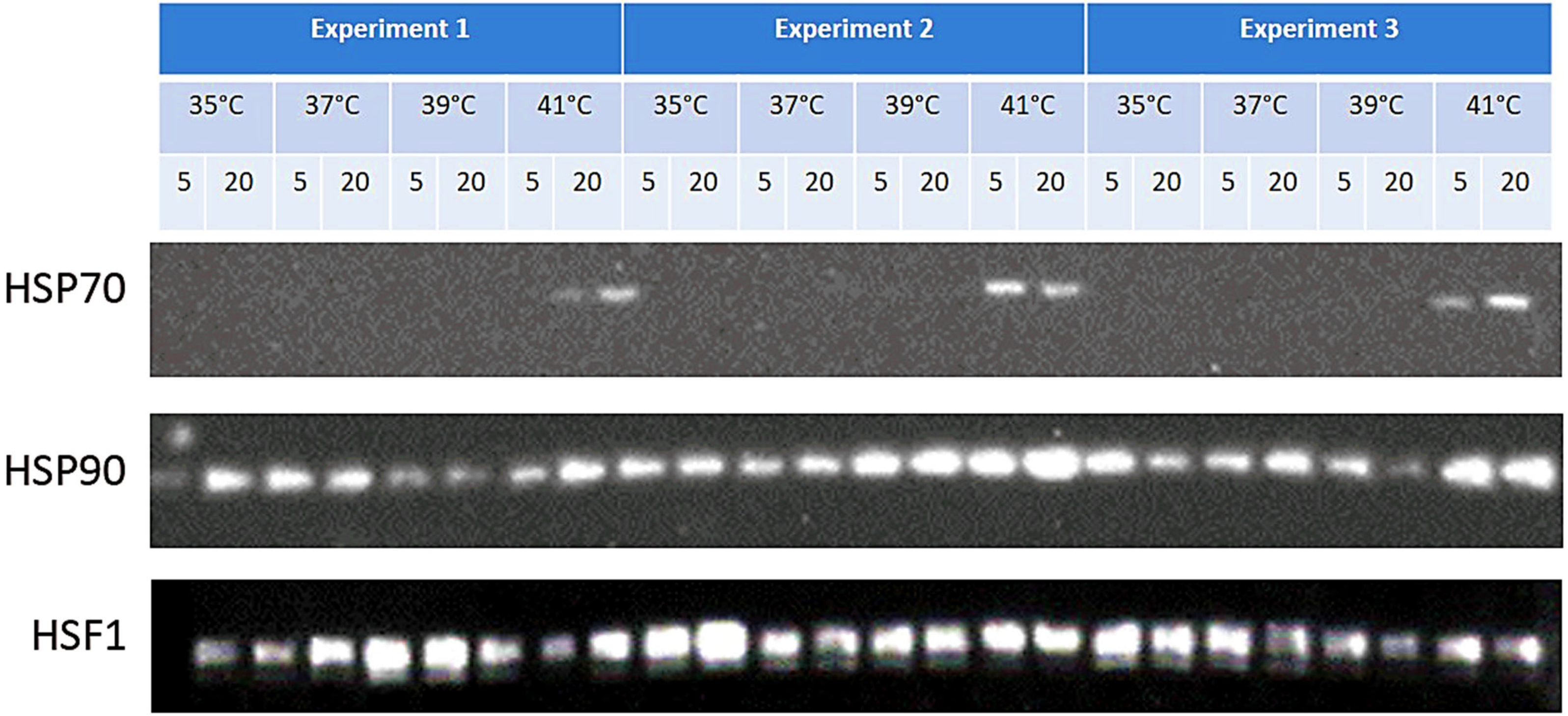


**Fig. S1** Overview of Western blot analysis of HSP70, HSP90 and HSF1 of myoblasts from pool 5 (5) or pool 20 (20) permanently cultured at 35°, 37°, 39° and 41°C. Experiment was performed three times (Experiment 1, 2, 3). For quality assurance the original blots are shown individually in Fig. S3 for HSP70, in Fig. S4 for HSP90 and in Fig. S5 for HSF1.

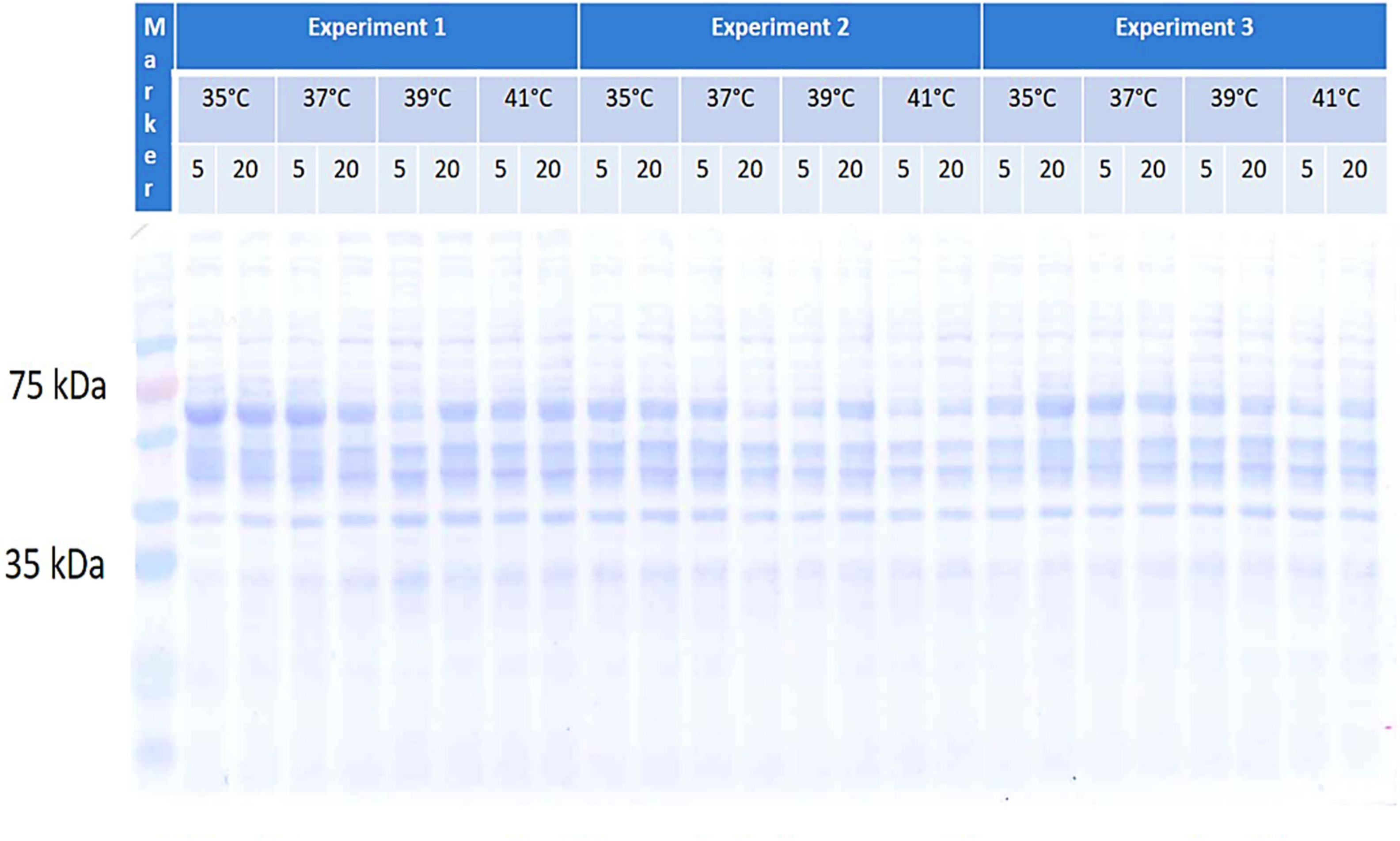


**Fig. S2** Coomassie blue loading control staining used for normalization of Western blot analysis (Fig. S1). Myoblasts from pool 5 (5) or pool 20 (20) were permanently cultured at 35°, 37°, 39° and 41°C. Experiment was performed three times (Experiment 1, 2, 3).

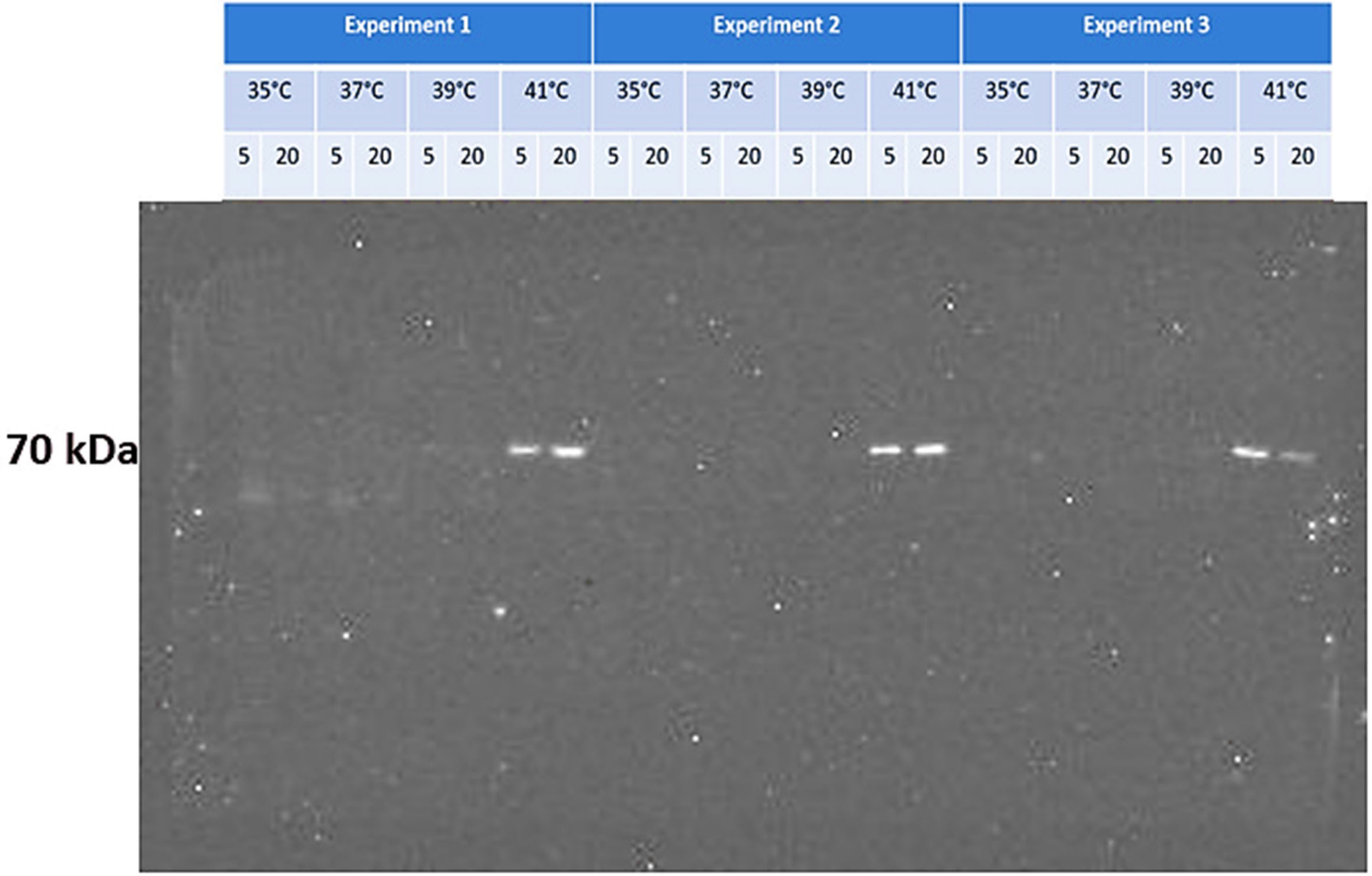


**Fig. S3** Original Western blot of HSP70 of myoblasts from pool 5 (5) or pool 20 (20) permanently cultured at 35°, 37°, 39° and 41°C. Experiment was performed three times (Experiment 1, 2, 3).

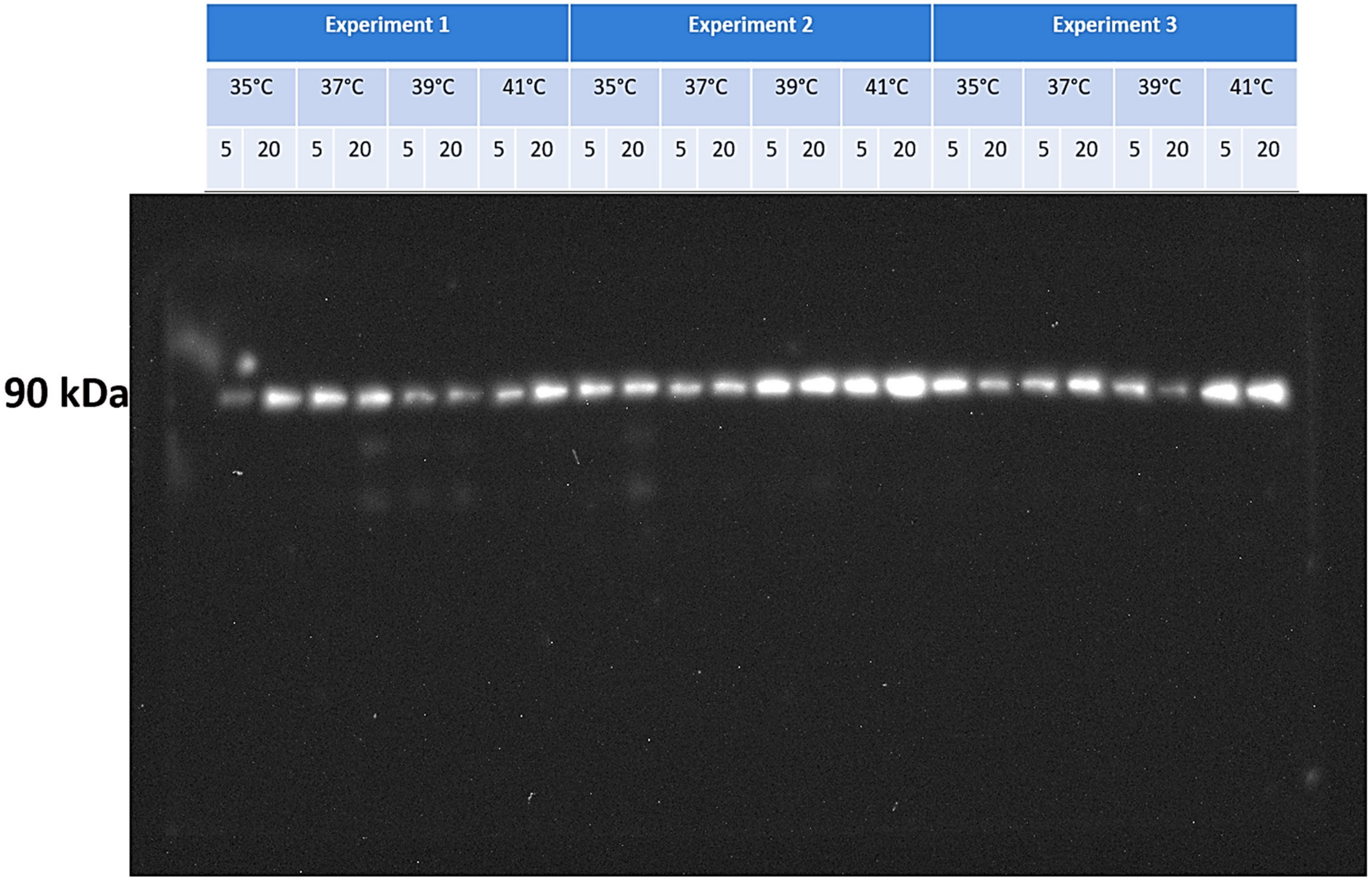


**Fig. S4** Original Western blot of HSP90 of myoblasts from pool 5 (5) or pool 20 (20) permanently cultured at 35°, 37°, 39° and 41°C. Experiment was performed three times (Experiment 1, 2, 3).

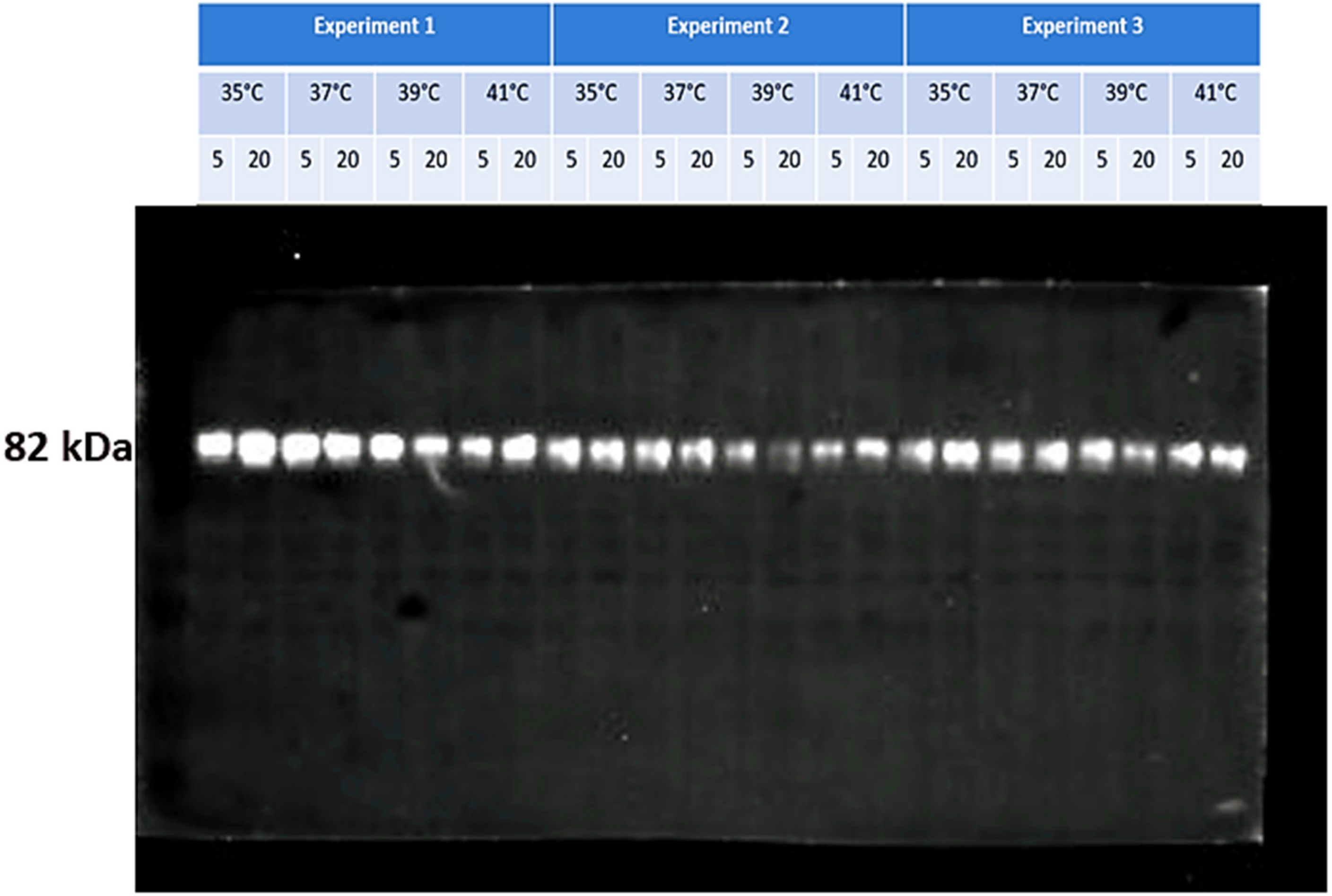


**Fig. S5** Original Western blot of HSF1 of myoblasts from pool 5 (5) or pool 20 (20) permanently cultured at 35°, 37°, 39° and 41°C. Experiment was performed three times (Experiment 1, 2, 3).

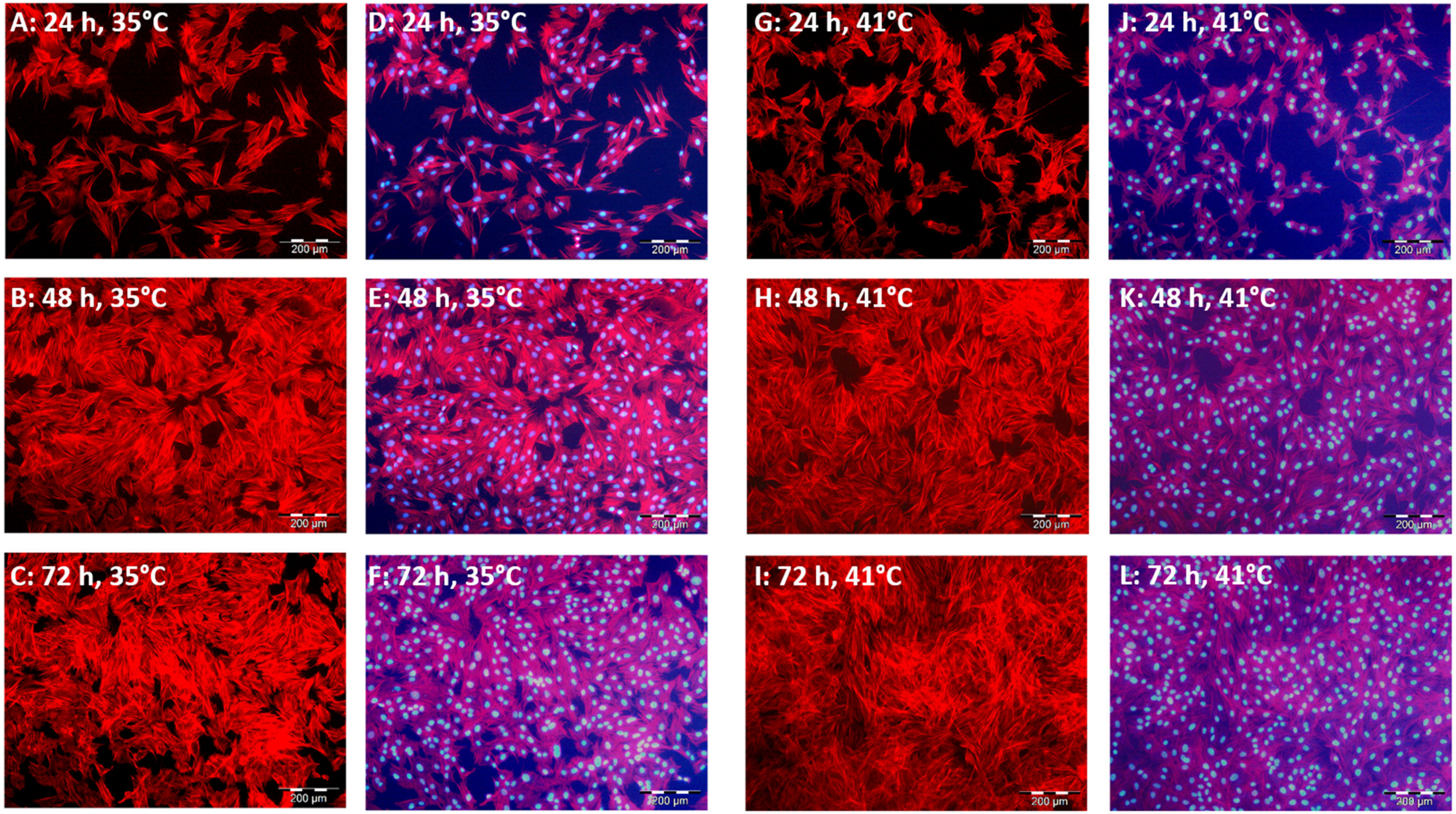


**Fig. S6** Myoblasts derived from satellite cells of *M. rhomboideus* of 5-day-old piglets were seeded on gelatin-coated dishes and cultivated at 35°C (A-F) or 41°C (G-L) for 24 h, 48 h and 72 h. A staining for actin filaments with Phalloidin CruzFluor™ 594 Conjugate (red: A-C, G-I) alone and an overlay with 4′,6-Diamidin-2-phenylindol (DAPI) for the nuclei (blue) were shown (D-F, J-L). Images were taken with Leica DM 2400 fluorescence microscope (Leica Microsystems, Wetzlar, Germany).
